# Enhancing cold tolerance through HubZIP6-mediated CBF activation and salicylic acid signaling in pitaya

**DOI:** 10.1186/s43897-026-00258-3

**Published:** 2026-09-03

**Authors:** Xinglong Hu, Irfan Ali Sabir, Zeliu Xu, Zhike Zhang, Jietang Zhao, Guibing Hu, Yonghua Qin

**Affiliations:** Guangdong Provincial Key Laboratory of Postharvest Science of Fruits and Vegetables and Key Laboratory of Biology and Genetic Improvement of Horticultural Crops (South China), Ministry of Agriculture and Rural Affairs, College of Horticulture, South China Agricultural University, Guangzhou, 510642 China

**Keywords:** Cold stress, HubZIP6, HuSABP2, Pitaya, Transcriptional regulation

## Abstract

Cold stress severely limits the yield and quality of fruit crops, yet its regulatory mechanisms in pitaya remain poorly understood. Here, we identified a cold-inducible bZIP transcription factor, HubZIP6, that plays a central role in enhancing cold tolerance in pitaya. HubZIP6 is a nuclear-localized protein with transcriptional activation activity, and its overexpression in Arabidopsis and tomato significantly improved cold tolerance, as reflected by higher survival rates, reduced ion leakage, and lower reactive oxygen species accumulation. Mechanistically, HubZIP6 directly binds to ACGT motifs in the promoters of *HuCBF1* and *HuCBF3*, thereby activating their expression under cold stress. In addition, HubZIP6 physically interacts with the salicylic acid-binding protein HuSABP2, which synergistically enhances the transcriptional activation of *HuCBF* genes. Notably, HubZIP6 also directly activates *HuSABP2*, forming a regulatory loop that connects CBF transcriptional control with salicylic acid signaling. Consistently, overexpression of *HuSABP2* further enhances cold tolerance in transgenic plants. Collectively, these findings demonstrate that cold tolerance is enhanced through HubZIP6-mediated integration of CBF activation and salicylic acid signaling, providing a promising genetic target for improving stress resilience in fruit crops.

**Supplementary Information:**

The online version contains supplementary material available at https://doi.org/10.1186/s43897-026-00258-3.

## Core

Cold stress inhibits the growth and yield of pitaya. *HubZIP6*, a cold-tolerance gene, enhances plant cold tolerance via the activation of *HuCBF1* and *HuCBF3* expression and HuSABP2-mediated salicylic acid (SA) signaling to coordinately regulate SA content and cold stress response in pitaya.

## Gene and accession numbers

*HubZIP6* (HU01G02772.1), *HuCBF1* (HU02G01866.1), *HuCBF3* (HU05G00587.1), and *HuSABP2* (HU06G00820.1).

## Introduction

Cold stress, which includes chilling stress (0 ℃−15 ℃) and freezing stress (< 0 ℃), is a significant abiotic stressor that adversely affects plant growth, development and agricultural production (Ding et al. 2024; Ding and Yang 2022; Guo et al. 2018). Chilling stress reduces the fluidity in the plant cell membrane, perturbs the protein homeostasis, and suppresses enzymatic activities (Kang et al. 2025; Qi et al. 2022). Freezing stress is instigated by the development of intracellular and extracellular ice crystals, which could result in irreversible cell damage and plant death in extreme situations (Kidokoro et al. 2022; Zhang et al. 2020). To quickly perceive and transmit cold signals, complex molecular regulatory networks and physiological metabolic mechanisms have evolved in plants (Mehrotra et al. 2020; Takahashi et al. 2024). DREB1/CBF (dehydration responsive element binding factor 1/C-repeat binding factor) transcription factors (TFs) are central regulators in the signaling of cold in plants, where they directly regulate the expression of downstream *COR* (cold regulated) genes under cold conditions (Jaglo-Ottosen et al. 1998; Liu et al. 2018a, b; Song et al. 2021). The CBF-COR pathway is well-known in other plant species, such as Arabidopsis (Liu et al. 1998, 2019), tomato (Wang et al. 2019), maize (Zeng et al. 2021) and apple (An et al. 2018, 2021; Yang et al. 2023). *MdCBFs* and *COR* genes are activated by abscisic acid (ABA) and calcium signaling pathways, contributing to cold tolerance (An et al. 2022; Wang et al. 2021)*.*

The *CBF* genes play critical roles in regulating plant responses to cold stress (Liu et al. 1998). Multiple TF families, including bHLH, MYB, NAC, and bZIP, have b een shown to directly influence *CBF* expression (Canibano et al. 2021; Feller et al. 2011; Mei et al. 2023; Xu et al. 2024; Yang et al. 2023). The bZIP family, one of the biggest and most functionally varied TFs in plants, has been shown to contribute to plant growth and development as well as tolerance to several abiotic stimuli, including cold, osmotic, drought, and salt stress (Fu et al. 2024; Li et al. 2022; van Gelderen et al. 2018; Yu et al. 2019). Plant bZIP TFs regulate gene expression by binding to specific *cis*-elements, especially those containing the ACGT core sequences, such as the G-box (CACGTG) and A-box (TACGTA) within target gene promoters (Yu et al. 2019). DgbZIP3 works with DgbZIP2 in chrysanthemums to regulate *DgPOD* expression, increase antioxidant enzyme activity, and modulate reactive oxygen species (ROS) levels, therefore improving cold stress tolerance (Bai et al. 2022). In contrast, TabZIP6 in wheat inhibits the expression of downstream *COR* genes by interacting with *CBF* genes, hence enhancing sensitivity to low temperatures and functioning as a negative regulator in the cold stress response (Cai et al. 2018). In apple, MdHY5 positively controls the transcription of *MdCBF1* by binding to the G-box element in the promoter, therefore affecting the expression of *CBF* and *COR* genes. Furthermore, MdHY5 also regulated the expression of CBF-independent *COR* genes. This indicates that MdHY5 enhances plant cold tolerance via both CBF-dependent and CBF-independent pathways (An et al. 2017). However, the function of bZIP TF in cold tolerance in pitaya remains unclear, and the regulatory relationship between bZIP and CBF is yet to be reported.

A large number of evidence indicate the pivotal roles of phytohormones, including ABA, JA (jasmonic acid), ethylene and SA (salicylic acid), in coordinating the responses of plants to environmental stresses through controlling signaling pathways related to stress responses (Gao et al. 2022; Gong et al. 2023, 2024; Ming et al. 2021). Although significant progress has been in understanding the roles of SA in plant immune responses, the mechanism of SA in the responses of plants to cold stress is less well understood (Liu et al. 2025; Wang et al. 2025). Recent studies have proven that SA can enhance plant cold tolerance by adjusting the expression of *COR* genes (Olate et al. 2018; Xiao et al. 2024c). SABP (SA-binding protein) is a vital enzyme that can help SA to activate the plant defense system, thereby increasing the activity of antioxidant enzymes and decreasing the accumulation of ROS to give rise to plant resistance development (Gong et al. 2023). In tobacco, *NtSABP2* functions as an ascorbate peroxidase (APX) or methyl salicylate (MeSA) esterase (Soares et al. 2022). SABP2-like esterase proteins can catalyze the demethylation of inactive MeSA to the active SA, which increases the plant resistance to contrary environmental condition (Wildermuth et al. 2002). Importantly, virus-induced gene silencing (VIGS) of the *HcSABP2* led to a decrease in drought resistance in kenaf seedlings (Zhang et al. 2025a, b). However, the regulatory mechanism of *SABP2* in pitaya under cold stress is still unknown.

Pitaya is a popular fruit for its satisfaction appearance, high nutritional value, and distinct taste. It has been rich in betalain, vitamins, antioxidants, minerals, dietary fiber, and it has cancer prevention effects as well (Shah et al. 2023). However, pitaya plants are highly vulnerable to cold stress. Cold stress not only causes reduced yield, decreased fruit quality and economic losses of pitaya, but also leads to the death of the plants in severe cases (Zhou et al. 2020). Therefore, understanding the response of the pitaya to cold stress is important to improve the quality and productivity of this fruit. Here, we have identified a cold-responsive TF, HubZIP6, which promotes the cold tolerance of genetically modified *Arabidopsis thaliana* and tomato plants. HubZIP6 induces the cold-induced expression of *HuCBF1*/*3* through direct promoter-interaction. Besides, HubZIP6 can bind with *HuSABP2* and promote the expression of *HuSABP2* to increase the cold resistance of transgenic plants. In addition, homozygous plants transformed with *HuSABP2* in *A. thaliana* and tomato also exhibit strong cold tolerance. In conclusion, the current investigation provide a new vision for the role of HubZIP6 in regulating the cold response in pitaya plants by both CBF-dependent and CBF-independent ways. These results have possible implications for the use of transgenic approaches in enhancing cold tolerance in pitaya.

## Results

### Gene cloning and characterization of the cold-responsive bZIP TF HubZIP6

**Fig. 1:**
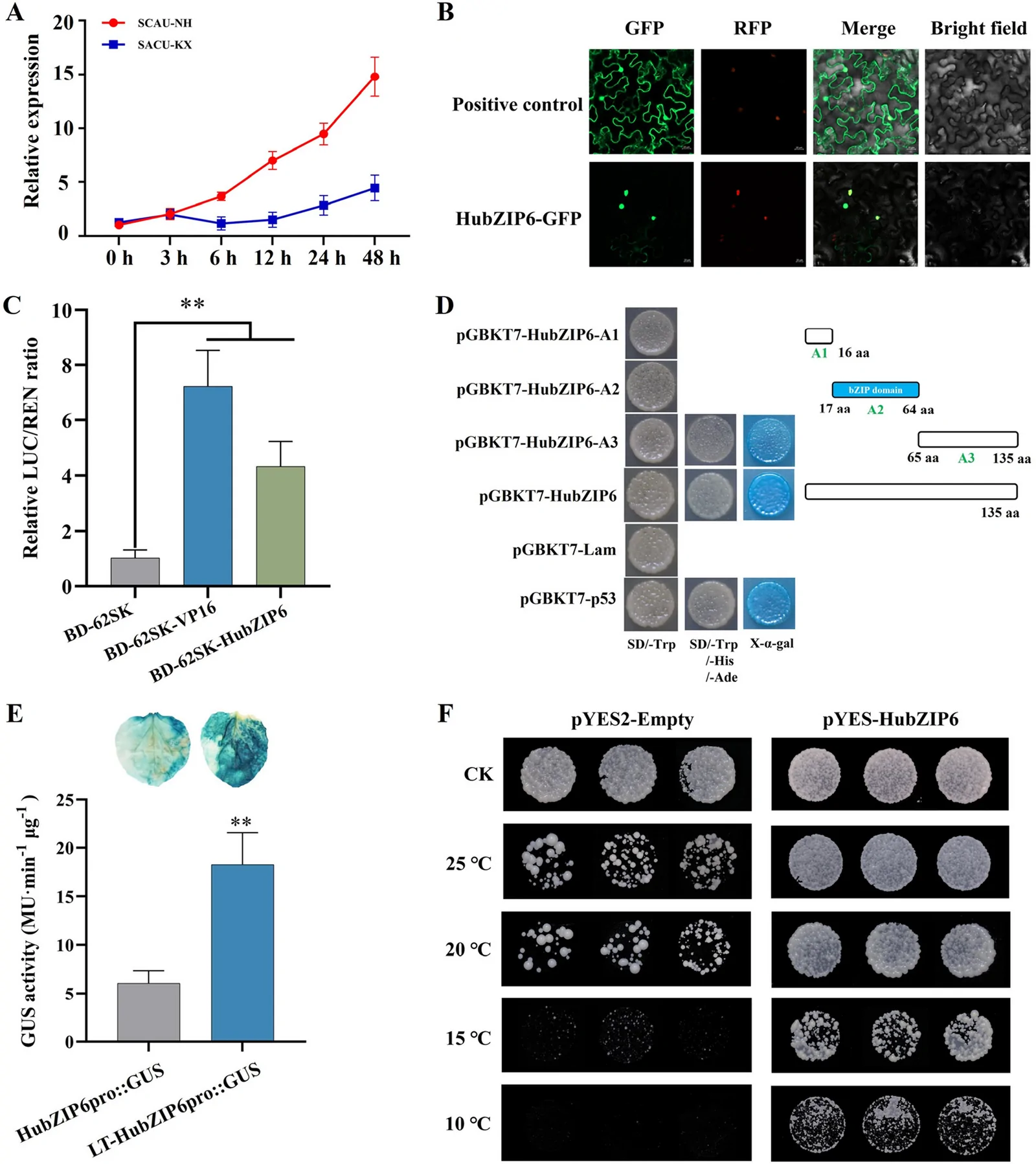
*HubZIP6* response to cold stress. **A** Relative expression levels of *HubZIP6* in two pitaya varieties under cold conditions (4 ℃). **B** Subcellular localization of HubZIP6 in *Nicotiana benthamiana* leaves (Bar = 20 μm). **C** Transcriptional activation of HubZIP6 in *N. benthamiana* leaves. **D** Transcriptional activation analyses of four fragments from HubZIP6 in yeast. **E** GUS staining analyses of the *HubZIP6* promoter in *N. benthamiana* leaves. **F** Verification of the *HubZIP6* function using a yeast system at different temperatures. The negative control is represented by pYES2-Empty, and each experiment was replicated three times. Error bars indicate the SD of three biological replicates. Significant differences were determined using Student’s *t*-test by comparison with CK (** *p* < 0.01)

Four points (0, 3, 6, and 12 h) of ‘SCAU-NH’ (resistant to low temperature) and ‘SCAU-KX’ (sensitive to low temperature) after treatment of 0, 3, 6, 12, 24, and 48 h at 4 ℃ were used for transcriptome sequencing (Accession number: PRJNA1335801). A total of 74 members of the *bZIP* gene family exhibited varying transcription levels in response to low-temperature treatment in pitaya (Figure S1A). To confirm the alteration in transcriptional levels at low temperatures, we selected several differentially expressed genes (DEGs) (exhibiting relatively high expression in ‘SCAU-NH’ and relatively low expression in ‘SCAU-KX’) and evaluated their expressions using qRT-PCR. The results aligned with those obtained from the transcriptome analysis (Figure S1B). Among them, in the cold-tolerant variety, ‘SCAU-NH’, there was a continuous increasing trend in the expression level of *HU01G02772.1*, compared with a relatively low level without significant change in the cold-sensitive variety of ‘SCAU-KX’ (Fig. 1A). We hypothesized that *HU01G02772.1* is important for cold response in pitaya and named it as *HubZIP6* according to its location on the chromosome (Figure S2). The complete cDNA sequence of *HubZIP6* contained a 408 bp open reading frame (ORF) encoding 135 amino acids.

Subcellular localization of HubZIP6 in the leaves of *N. benthamiana* showed that HubZIP6 was localized in the nucleus (Fig. 1B). Trans-activation of HubZIP6 was analyzed in *N. benthamiana* leaves by the dual-luciferase reporter assay. Compared with the negative control, positive control (BD-62SK-VP16) and HubZIP6 had a higher LUC/REN ratio, which reflects transcriptional activation ability of HubZIP6 (Fig. 1C). Furthermore, we used the yeast two-hybrid system to clarify the transcriptional activation region of HubZIP6. The experimental results showed that the yeast associated with transformation grew normally using either the full-length HubZIP6 ORF sequence ranging from 1 to 135 amino acids or the HubZIP6 C-terminal sequence ranging from 65 to 135 amino acids, while the HubZIP6 N-terminal sequence ranging from 1 to 64 amino acids did not show normal growth (Fig. 1D). Those results indicated that the polypeptide region 65–135 is the transcriptional activation region of HubZIP6. The 2000 bp promoter region of *HubZIP6* was cloned into a GUS reporter vector for further analysis. According to GUS staining, higher activity of the *HubZIP6* promoter was detected under low-temperature stress conditions, which was consistent with the result of GUS enzyme activity (Fig. 1E). In addition, a system of yeast was used to further verify the cold tolerance of the HubZIP6. Yeast strains expressing the HubZIP6 exhibited robust growth under low-temperature conditions, whereas yeast strains containing the empty plasmid demonstrated inhibited growth (Fig. 1F).

### Overexpressing *HubZIP6 *improves cold tolerance in transgenic *Arabidopsis* plants

**Fig. 2:**
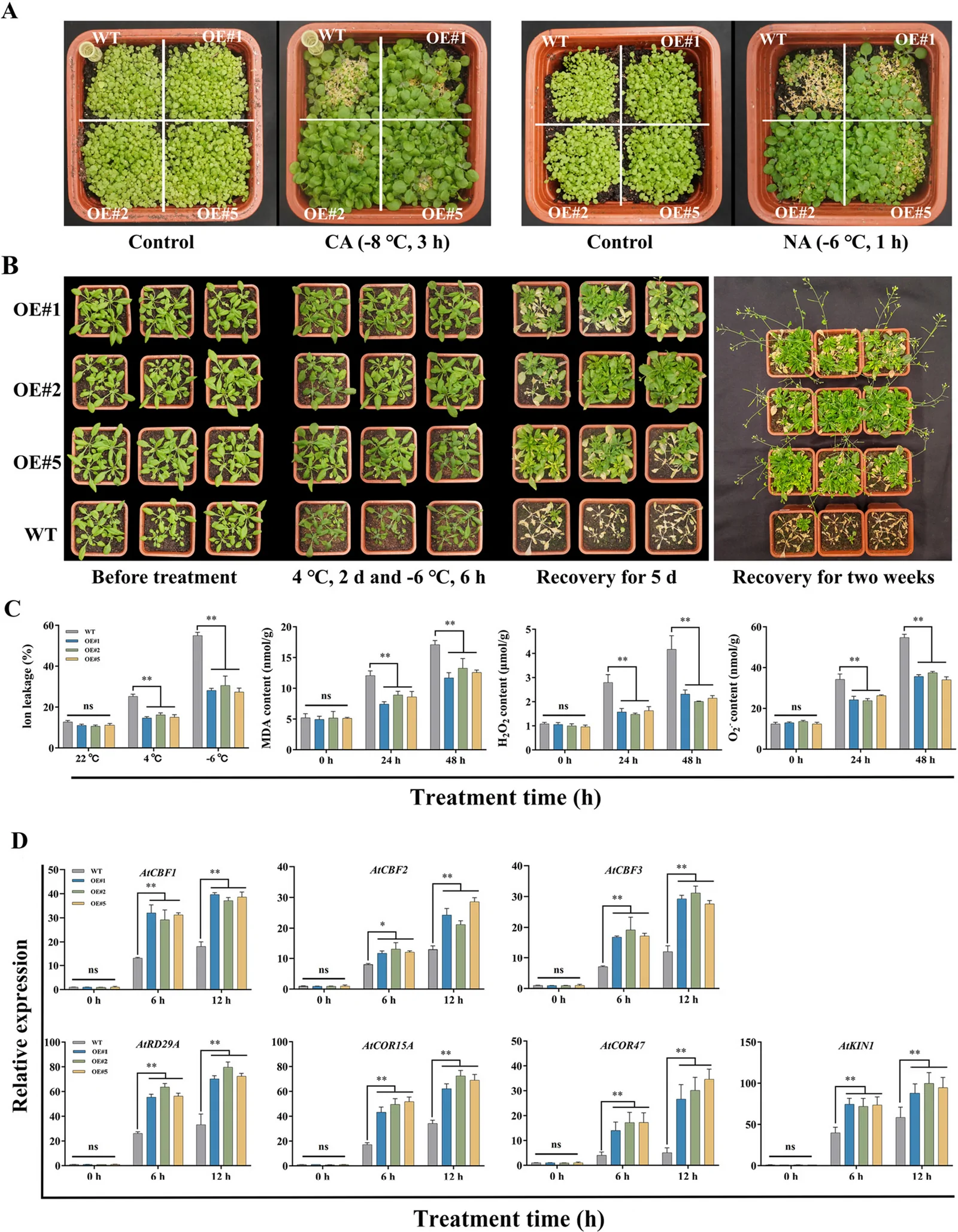
Overexpressing *HubZIP6* in *Arabidopsis* significantly enhances cold tolerance. **A** The transgenic *Arabidopsis* exhibits freezing tolerance under both non-acclimated (NA) and cold-acclimated (CA) conditions. **B** Phenotypic comparison of 4-week-old transgenic and wild-type (WT) *Arabidopsis* after cold treatment. **C** Assessment of ion leakage, MDA content, H_2_O_2_, and O_2_^.−^ levels in WT and transgenic plants after cold stress. **D** Expression analyses of *AtCBFs* and cold-responsive genes in WT and transgenic lines under cold stress conditions. Error bars indicate the SD of three biological replicates. Significant differences were determined using Student’s *t*-test by comparison with CK (*p < 0.05, ** *p* < 0.01). ns stands for no significant difference

Genetic transformation was performed on *A. thaliana* to explore the function of *HubZIP6*. Following screening on 1/2MS medium supplemented with 100 mg/L Kan, eight homozygous lines of transgenic *A. thaliana* plants were obtained. qRT-PCR was used to ascertain the *HubZIP6* expression levels in these homozygous transgenic lines. Three transgenic lines (OE-1, OE-2, and OE-5) with rather high expression of *HubZIP6* were chosen for cold tolerance measurement (Figure S3). One-week-old seedlings used for cold treatment were named NA (non-cold-acclimated) and CA (cold-acclimated) groups. Compared with wild-type (WT) plants, the transgenic seedlings exhibited improvement in freezing tolerance under both NA and CA conditions, and significantly higher survival rates were observed for the transgenic lines than the WT plants (Fig. 2A).

To explore the role of *HubZIP6* in regulating cold tolerance in *Arabidopsis*, 4-week seedlings were subjected to freezing after a 2 d cold hardening period. At normal temperature conditions, no difference was observed between the transgenic lines, mutant and WT plants. After low temperature treatment (4 ℃ for 2 d and −6 ℃ for 6 h) and a subsequent of 5 d recovery at 22 ℃, transgenic plants exhibited obviously cold-tolerance performance compared with WT plants, while the mutant lines exhibited significantly reduced cold tolerance (Fig. 2B and Figure S4). Most transgenic plants were able to recover from growth and even bolt and flower after cold stress. Leaf samples were collected from all transgenic, mutant and WT lines for the determination of stress-related metabolites after cold treatment. Overexpression of *HubZIP6* led to a significant decrease in ion leakage and MDA contents and ROS levels under cold stress compared with WT plants, while an opposite trend was observed in the mutants (Fig. 2C and Figure S4). To further elucidate its function, the expression of *Arabidopsis COR* genes including *AtCBF1*, *AtCBF2*, *AtCBF3*, and *AtCOR47* were examined in transgenic, mutant and WT plants after cold treatment. Compared with WT plants, the expression levels of the four *COR* genes were highly induced in *HubZIP6*-overexpressing lines, but significantly down-regulated in the mutant lines (Figure S4). These findings indicated that *HubZIP6* enhances cold tolerance in *Arabidopsis* by positively regulating the expression of *CBF* and *COR* genes while reducing ion accumulation, MDA and ROS levels.

### *HubZIP6* enhances the cold tolerance of transgenic tomato plants

**Fig. 3:**
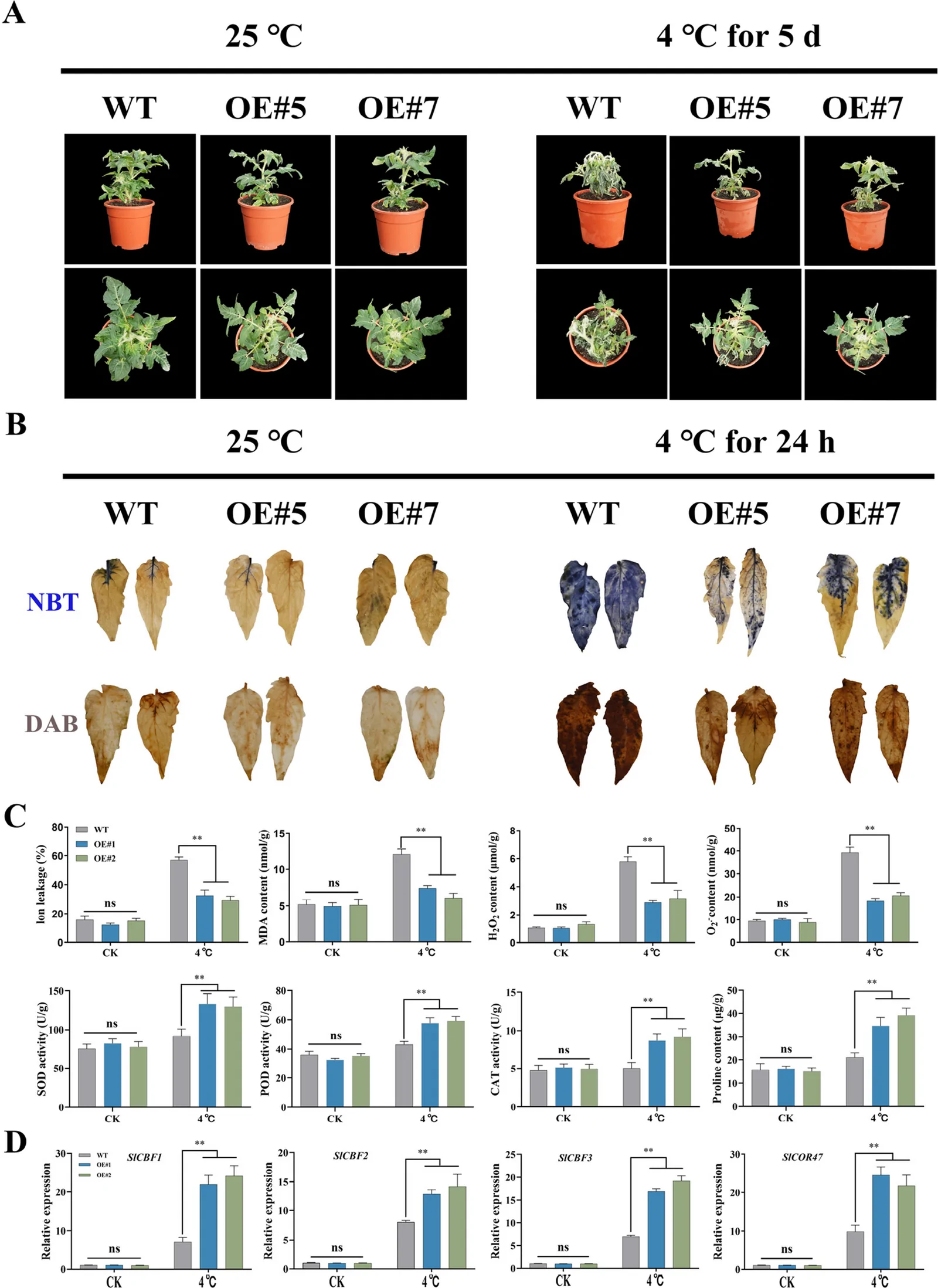
*HubZIP6* enhances the cold tolerance of transgenic tomato plants. **A** Phenotypic comparison of transgenic and WT tomato plants after cold treatment. **B** NBT and DAB staining. **C** Changes of ion leakage, MDA content, H_2_O_2_, O_2_^.−^, SOD, POD, CAT, proline content, in WT and transgenic plants under cold stress. **D** Expression analyses of *SlCBFs* and *SlCOR47* in WT and transgenic plants under cold stress. Error bars indicate the SD of three biological replicates. Significant differences were determined using Student’s *t*-test by comparison with CK (** *p* < 0.01). ns stands for no significant difference

To further assess *HubZIP6* role, we overexpressed *HubZIP6* in tomato plants to generate T_1_ transgenic lines. Two T_2_ transgenic lines, OE#5 and OE#7, displaying elevated *HubZIP6* expression levels, were chosen for further investigation (Figure S5). After 5 d of treatment at 4 ℃, most leaves of WT plants exhibited shrinkage and drooping, while the transgenic tomato plants overexpressing *HubZIP6* could grow normally, with only a few wilting leaves (Fig. 3A). To investigate the levels of H_2_O_2_ and O_2_^.−^ accumulation, DAB and NBT staining were conducted following a 24 h exposure to 4 ℃. Under normal condition, there was no significant difference in leaf staining between transgenic tomato plants and WT. After cold treatment, DAB and NBT staining revealed extensive dark brown and dark blue patches on WT plant leaves, respectively. In contrast, transgenic plant leaves exhibited significantly fewer brown and blue spots (Fig. 3B), indicating lower ROS levels in transgenic plants after cold treatment. Quantitative analyses of H_2_O_2_ and O_2_^.−^ contents corroborated the staining results (Fig. 3C). Meanwhile, significantly lower MDA content and ion leakage rates in the transgenic lines were detected compared with the WT plants (Fig. 3C). Additionally, there were no significant difference in the activities of SOD, POD, CAT, and proline content between the transgenic and WT lines under normal temperature conditions. Following cold treatment, the activities of these enzymes and proline content were significantly increased in the *HubZIP6* transgenic lines compared with the WT plants (Fig. 3C). The expression levels of *SlCBF1*/*2*/*3* and *SlCOR47* were significantly higher in the two transgenic lines compared with the WT plants (Fig. 3D). These findings indicated that *HubZIP6* positively influences *CBFs* and *COR* genes, increases the activities of SOD, POD, and CAT, and reduces ROS levels, thereby enhancing cold tolerance in transgenic tomato.

### HubZIP6 activates the transcription of *HuCBF1* and *HuCBF3* by directly binding to their promoters

The CBF-COR pathway plays a vital role in responding to cold stress (Liu et al. 2019). This pathway regulates various physiological and biochemical processes. TFs interact with *CBF* promoters, influencing their expressions and thus contributing to the cold stress response (Yu et al. 2019). To explore the role of HubZIP6 in the CBF regulatory pathway, we performed a CBF homology search in the pitaya genome database using *Arabidopsis* CBF protein sequences as queries. Three pitaya CBF homologs: *HuCBF1* (HU02G01866.1), *HuCBF2* (HU04G00141.1), and *HuCBF3* (HU05G00587.1) were identified. The Plant-Care online database was used to predict conserved *cis*-acting elements in the promoters of *HuCBF1*, *HuCBF2*, and *HuCBF3*, all of which had the bZIP binding site ACGT-motif (Supplementary Tables).

**Fig. 4:**
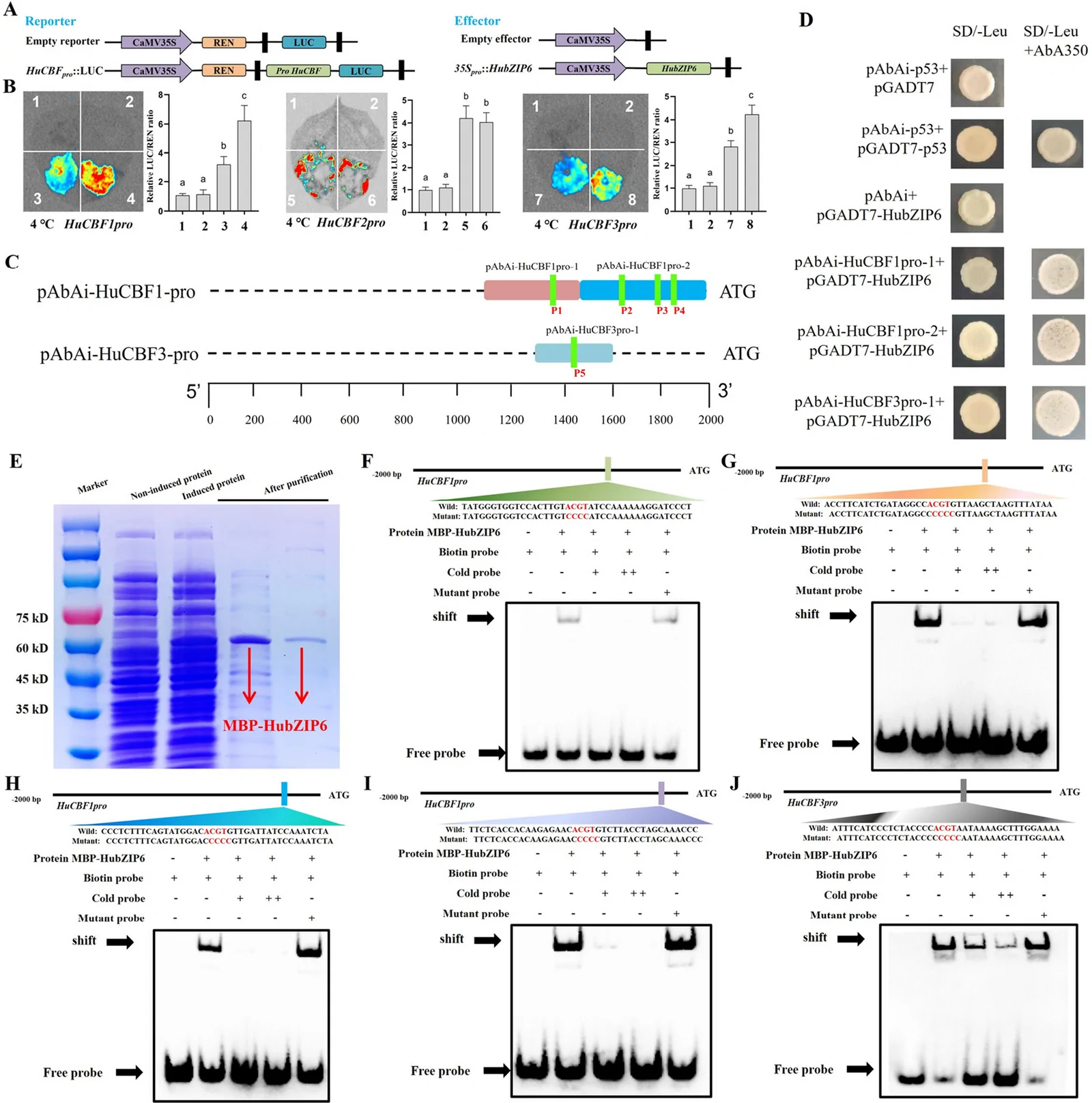
HubZIP6 enhances the expressions of *HuCBF1*/*3* during cold treatment*.* **A** Schematic diagram of the reporter and effector vectors. **B** Fluorescence observations and LUC/REN ratio of the dual-luciferase assay. 1, empty reporter and effector; 2, empty reporter +35S::HubZIP6; 3, proHuCBF1::LUC + empty effector; 4, proHuCBF1::LUC +35S::HubZIP6; 5, proHuCBF2::LUC + empty effector; 6, proHuCBF2::LUC +35S::HubZIP6; 7, proHuCBF3::LUC + empty effector; and 8, proHuCBF3::LUC +35S::HubZIP6. **C** Schematic diagram of the location of the G-box on the promoters. The green rectangle (P1-P5) represents the five probes (G-box) used in the EMSA, and pAbAi-HuCBF1pro-1/2 and pAbAi-HuCBF3pro-1 represent the bait yeast promoter fragments used in the Y1H. **D** Y1H assays between HubZIP6 and *HuCBF1*/*3*. **E** Results of induction and purification of HubZIP6 protein. **F**-**J** Verification of the binding ability between HubZIP6 and the five probes from *HuCBF1*/*3* promoters by competitive and mutant EMSA. Error bars indicate the SD of three biological replicates. Different letters indicate significant differences (One-way ANOVA, Tukey’s test, *p* < 0.05)

The interactions between HubZIP6 and the three *HuCBFs* promoters were analyzed using the dual-luciferase reporter (DLR) system (Fig. 4A). Compared with no fluorescence for co-expression of the empty vectors, co-expression of either the HuCBF1pro::LUC or HuCBF3pro::LUC construct with the empty effector vector showed a noticeable fluorescence signal after cold treatment, which was significantly enhanced by the presence of HubZIP6 protein. However, HubZIP6 does not activate the *HuCBF2* promoter, indicating specificity in its regulatory function (Fig. 4B). The increased LUC/REN ratio suggested that *HubZIP6* functions as a positive regulator for *HuCBF1*/*HuCBF3* and activates their expressions under cold stress.

Y1H assays were performed to identify the connection between HubZIP6 and the *HuCBF1*/*HuCBF3* promoters (Fig. 4C). Yeast cells harboring the pGADT7-HubZIP6 vector along with the HuCBF1/HuCBF3 pro-bait vector exhibited robust growth on selective medium, compared with no growth for control strains transformed with the empty pGADT7 vector (Fig. 4D). The findings indicated that HubZIP6 can bind the promoters of *HuCBF1* and *HuCBF3*. EMSA was conducted to identify the direct binding of the HubZIP6 protein to the promoters of *HuCBF1* and *HuCBF3*. The HubZIP6 protein was induced and purified (Fig. 4E). The promoters of *HuCBF1* and *HuCBF3* contained four and one bZIP TF binding site (ACGT motifs), respectively (Fig. 4C). Probes labeled with biotin were synthesized using the flanking sequences of the identified sites (primers were detailed in Supplementary File S2). Results from EMSA indicated the binding of HubZIP6 protein to specific sites within the promoters of *HuCBF1* and *HuCBF3* (Fig. 4F-J). Competition with unlabeled probes led to a notable decrease in band intensity, whereas mutation of the core recognition element sequence resulted in the restoration of band intensity, thereby demonstrating the particularity of HubZIP6 protein binding to these binding sites (Fig. 4F-J).

### The HubZIP6-HuSABP2 interaction promotes the binding ability of HubZIP6 on *HuCBF1 *and *HUCBF3* promotes

**Fig. 5:**
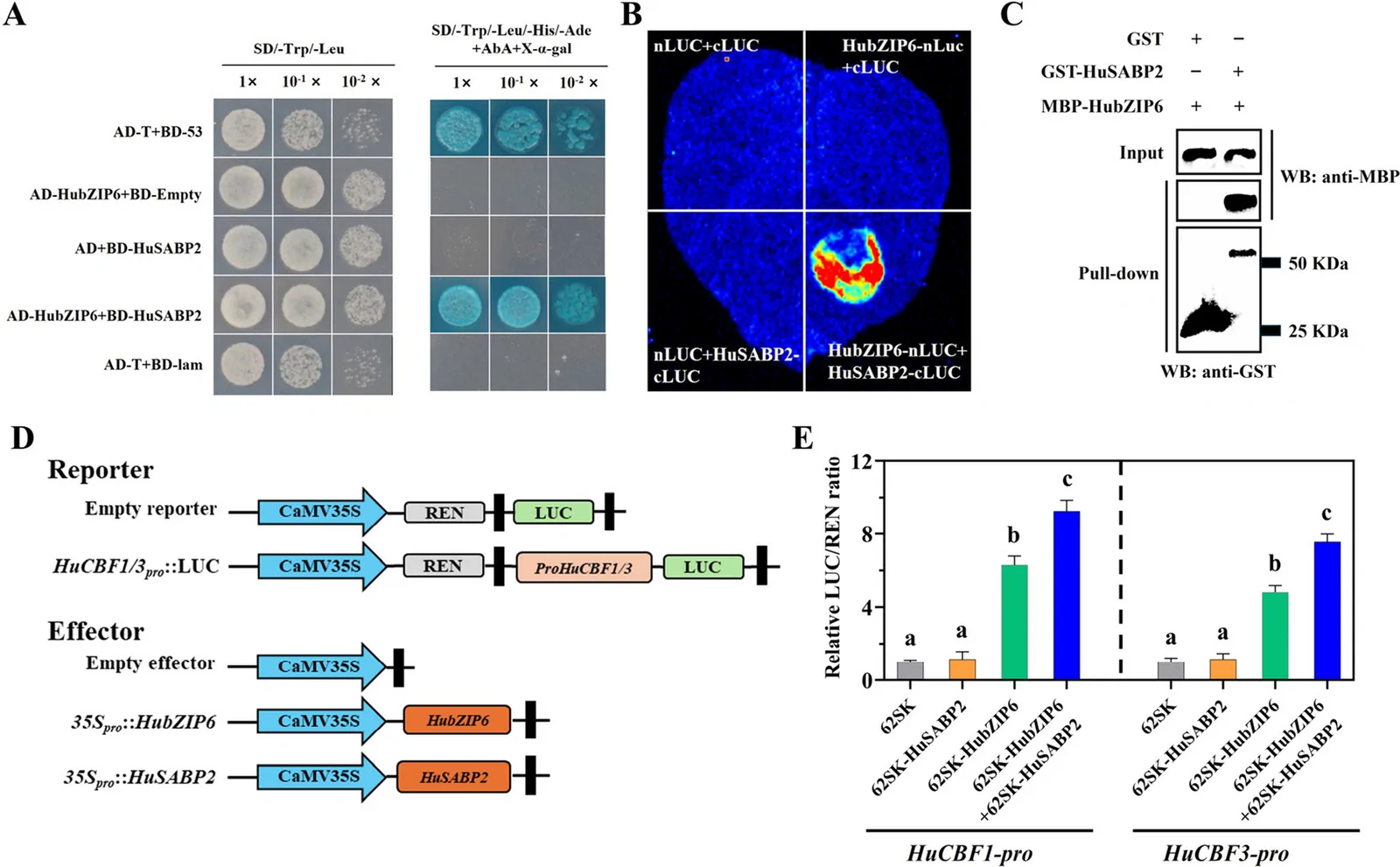
HubZIP6 interacts with HuSABP2 and promotes its expression. **A** Y2H analyses of the interaction between HubZIP6 and HuSABP2. AD-T + BD-53 and AD-T + BD-lam were used as positive and negative controls, respectively. The positive yeast was stained using X-α-Gal. **B** LCI analysis of the interaction between HubZIP6 and HuSABP2. HubZIP6 and HuSABP2 were separately fused to the N-terminus or C-terminus of luciferease (LUC), and the resulting constructs (nLUC-HubZIP6, cLUC-HuSABP2) were co-infiltrated in *N. benthamiana* leaves. LUC illuminance was observed under the in vivo plant imaging system. **C** Determination of interaction between HubZIP6 and HuSABP2 by GST pull-down assay. Recombinant GST-HuSABP2 bound to the GST Sepharose beads was incubated with MBP-HubZIP6. The eluted proteins were detected by immunoblotting using anti-GST and anti-MBP antibodies, respectively. **D** Schematic diagrams of *HuCBF1*/*3* promoter and the schematic diagrams of effector and reporter constructs used for dual luciferase (LUC) transient expression assay. **E** LUC/REN ratios of *N. benthamiana* leaves infiltrated with HubZIP6 and HuSABP2, alone or together, and *HuCBF1*/*3* promoters were used to construct reporter vector. Error bars indicate the SD of three biological replicates. Different letters indicate significant differences (One-way ANOVA, Tukey’s test, *p* < 0.05)

The potential CBF-independent pathways regulated by HubZIP6 were investigated by screening a Y2H system for potential interacting proteins. *HuSABP2* was identified as a yeast cell-interacting partner of HubZIP6. Y2H assays confirmed the interaction between HubZIP6 and HuSABP2. Yeast cells co-transformed with AD-HubZIP6 and BD-HuSABP2 displayed robust growth on SD/-Trp/-Leu/-His/-Ade and activated X-α-gal activity (Fig. 5A). LCI assays showed that LUC activity was strongly reconstituted in the *N. benthamiana* leaves co-expressing HubZIP6-nLUC and HuSABP2-cLUC (Fig. 5B), suggesting that HubZIP6 interacted with HuSABP2. In addition, GST pull-down assays indicated that GST-HuSABP2 could pull down MBP-HubZIP6, but not GST alone, implying the direct interaction between HubZIP6 and HuSABP2 *in vitro* (Fig. 5C). These results suggested that HubZIP6 could interact with HuSABP2. The effects of protein interaction on HubZIP6-mediated activation of downstream target genes were investigated by DLR assays (Fig. 5D). Compared with the expression of 62SK-HubZIP6 alone, co-expression of 62SK-HubZIP6 and 62SK-HuSABP2 led to a greater activation of the LUC reporter gene driven by the promoters of *HuCBF1* and *HuCBF3* (Fig. 5E), implying that the interaction between HubZIP6 and HuSABP2 enhanced the activation activity of HubZIP6.

### HubZIP6 activates the transcription of *HuSABP2* by directly binding to its promoter

**Fig. 6:**
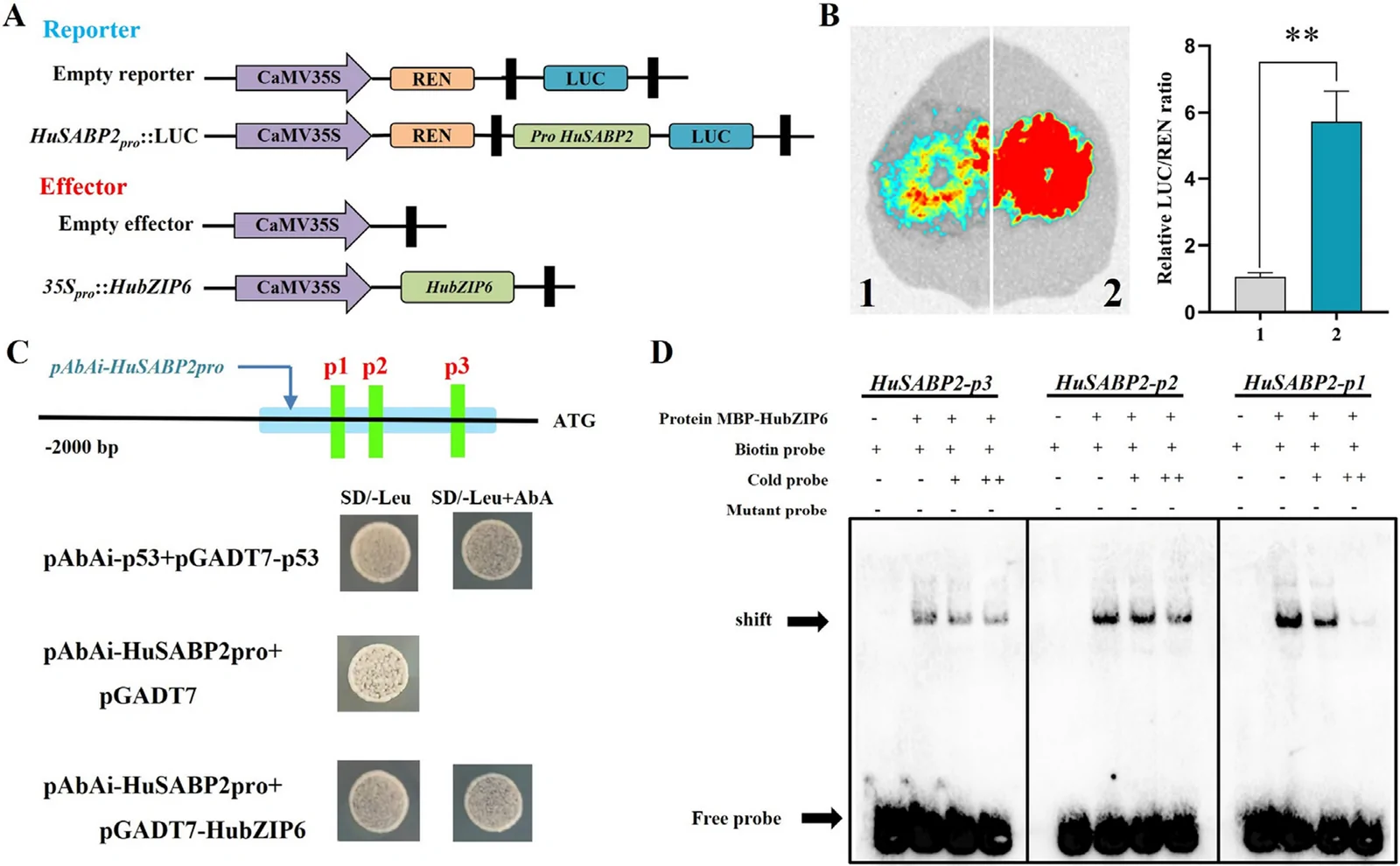
**A** Schematic diagram of the reporter and effector vectors. **B** Fluorescence observations and LUC/REN ratio of the dual-luciferase assay. 1, proHuSABP2::LUC + empty effector; 2, proHuSABP2::LUC +35S::HubZIP6. **C** Y1H assays between HubZIP6 and the promoters of *HuSABP2.* pAbAi-p53 + pGADT7-p53 and pAbAi-HuSABP2pro + pGADT7 were used as positive and negative controls, respectively. The green rectangle (p1-p3) represents the three probes (G-box) used for EMSA (**D**). Error bars indicate the SD of three biological replicates. Significant differences were determined using Student’s *t*-test by comparison with CK (** *p* < 0.01)

Results from Y1H, EMSA and DLR assays showed that HubZIP6 directly binds the *HuSABP2* promoter and therefore activates its expression. Co-expression of the construct HuSABP2pro::LUC with an empty effector vector resulted in a basal fluorescence signal upon cold treatment while the fluorescence signal was stimulated markedly in the presence of HubZIP6 (Fig. 6A-B). Yeast cells co-transformed with pGADT7-HubZIP6 and HuSABP2pro-bait (the positive control) appeared healthy on the screening medium, but control (containing empty pGADT7 vectors) did not grow (Fig. 6C). Promoter analysis of the 2000 bp upstream region of *HuSABP2* revealed three ACGT motifs, which are consensus binding sites for bZIP TFs. EMSA demonstrated that HubZIP6 specifically binds at all three sites (Fig. 6D). Collectively, these findings suggested that HubZIP6 interacts with *HuSABP2* and increases its transcription through its direct interaction with the promoter.

### *HuSABP2* is responsive to low-temperature stress

**Fig. 7:**
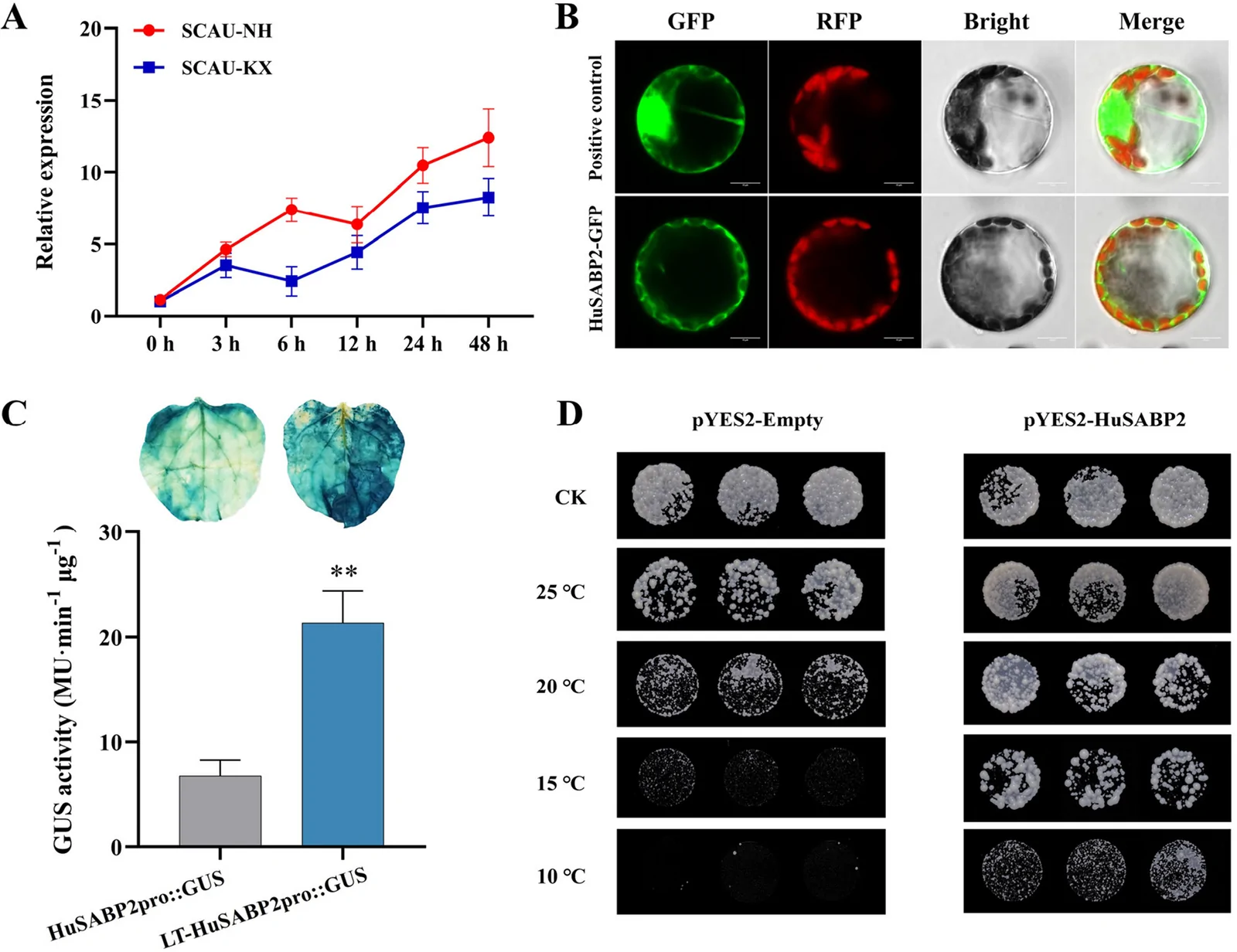
Response of *HuSABP2* to cold stress. **A** Relative expression levels of *HuSABP2* in two pitaya varieties under cold conditions (4 ℃). **B** Subcellular localization of HuSABP2 in *N. benthamiana* leaves. Bars = 20 μm. **C** GUS staining and GUS enzyme activity analyses of the *HuSABP2* promoter in *N. benthamiana* leaves. **D** Verification of the cold tolerance of HuSABP2 by the yeast system. pYES2-Empty represents the negative control. Each experiment was repeated three times. Error bars indicate the SD of three biological replicates. Significant differences were determined using Student’s *t*-test by comparison to CK (** *p* < 0.01)

Bioinformatics and expression profile analyses were conducted to identify the function of *HuSABP2*. The full-length cDNA sequence of *HuSABP2* consisted of an ORF 795 bp, which encoded 264 amino acid. The expression level of *HuSABP2* in the two pitaya genotypes was continuously increasing during cold treatment (Fig. 7A). The subcellular localization showed the localization of HuSABP2 in the cytoplasm (Fig. 7B). The GUS reporter vector was cloned with the *HuSABP2* promoter (2000 bp). Cold stress increased the *HuSABP2* promoter activity under low temperature, as observed through the GUS staining and GUS enzyme activity assay (Fig. 7C), respectively. Subsequent confirmation of avoidance of chilling-induced cell death through the involvement of *HuSABP2* in cold tolerance was performed using a yeast system. At 15 ℃ and 10 ℃, yeast expressing HuSABP2 displayed growth, whereas the negative control displayed significant abnormalities or absence of growth (Fig. 7D).

### Overexpressing *HuSABP2* enhances cold tolerance in transgenic *Arabidopsis* plants

**Fig. 8:**
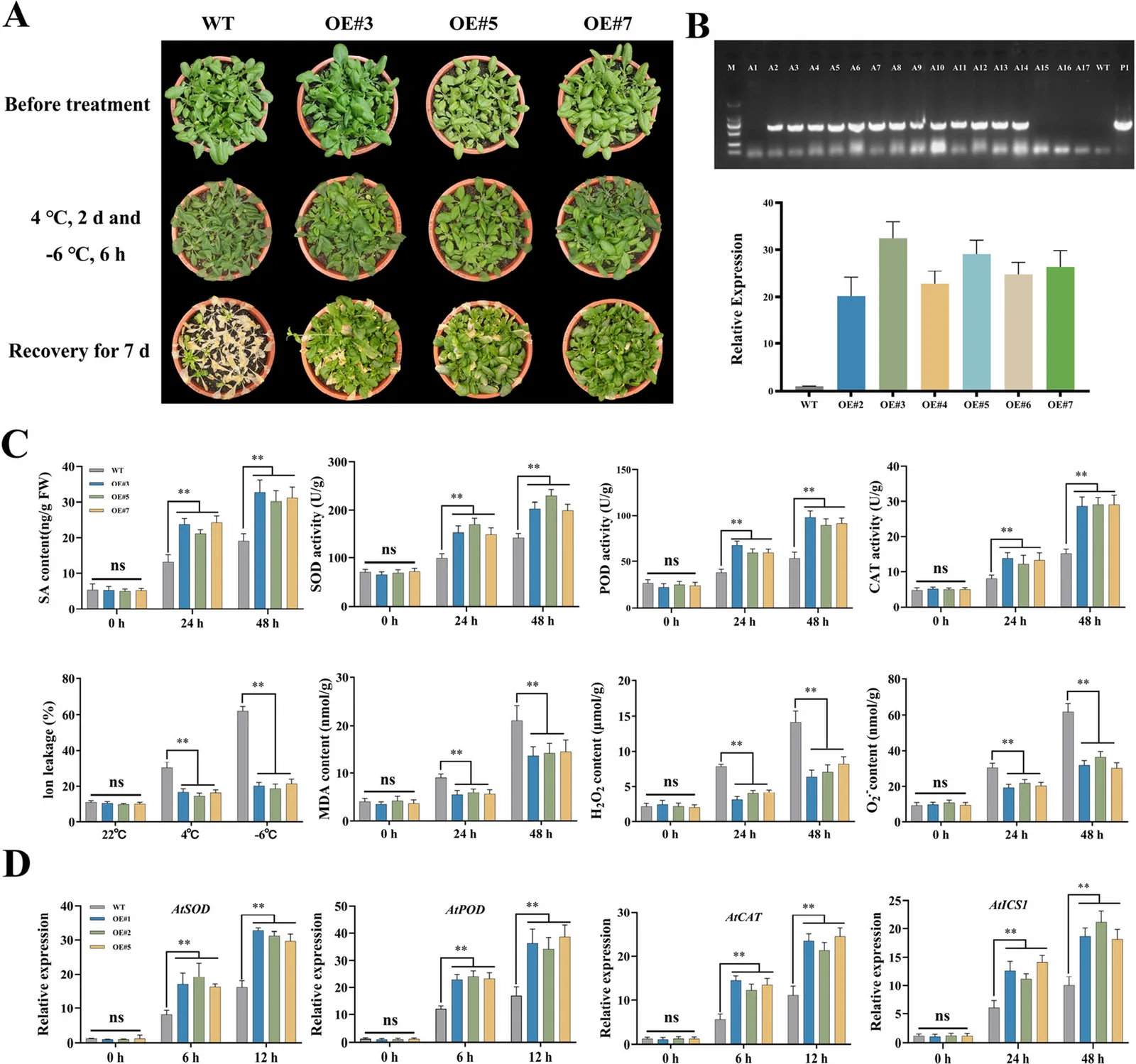
Overexpressing *HuSABP2* enhances cold tolerance in transgenic *Arabidopsis* plants. **A** Phenotypic comparison of 4-week-old transgenic and WT *Arabidopsis* after cold treatment. **B** Identification of transgenic *Arabidopsis* lines by PCR. M: DNA marker 2000; A1-A17: *Arabidopsis* transformed with the *HuSABP2* gene; P1: positive control (pPZP6K90-HuSABP2). Expression levels of *HuSABP2* in WT and transgenic plants. **C**-**D** Changes of SA, SOD, POD, CAT, proline content, ion leakage, MDA and ROS in WT and transgenic *Arabidopsis* under cold stress. **D** Expression levels of *AtSOD*, *AtPOD*, *AtCAT* and *AtICS1* in WT and transgenic *Arabidopsis* under cold stress. Error bars indicate the SD of three biological replicates. Significant differences were determined using Student’s *t*-test by comparison with CK (** *p* < 0.01). ns stands for no significant difference

The cold tolerance function of *HuSABP2* was investigated by the production of transgenic *A. thaliana* plants overexpressing *HuSABP2* gene in the T_3_ generation. PCR and qRT-PCR analyses revealed the existence of three independent lines (OE#3, OE#5, and OE#7) exhibiting high expression levels of *HuSABP2* that were selected for the freezing tolerance analyses (Fig. 8B). Compared with WT plants, overexpression of *HuSABP2* significantly enhanced cold tolerance, whereas the mutant lines were almost wilted or dead after freezing treatment (Fig. 8A and Figure S7). SA content, SOD, POD and CAT activities were significantly increased in transgenic lines, whereas opposite changes of SA level were observed in the mutant lines (Fig. 8C and Figure S7). Determination of ions leakage, MDA, and ROS levels revealed that the membrane injury was lower in transgenic plants compared with WT under cold stress, while ions leakage was significantly increased in the mutant lines (Fig. 8C and Figure S7). Compared with WT plants, the expressions of the antioxidant enzyme genes (*AtSOD*, *AtPOD* and *AtCAT*) and SA biosynthetic gene (*AtICS1*) were highly upregulated, as in the physiological response observed in overexpressing *HuSABP2* lines, while significantly down-regulated in the mutant lines (Fig. 8D and Figure S7). These findings suggested the role of *HuSABP2* in stimulating cold tolerance in *Arabidopsis* through the SA signaling pathway and reinforcement of antioxidant defense both at the enzymatic and transcriptional levels.

### *HuSABP2* enhances the cold tolerance of transgenic tomato plants

**Fig. 9:**
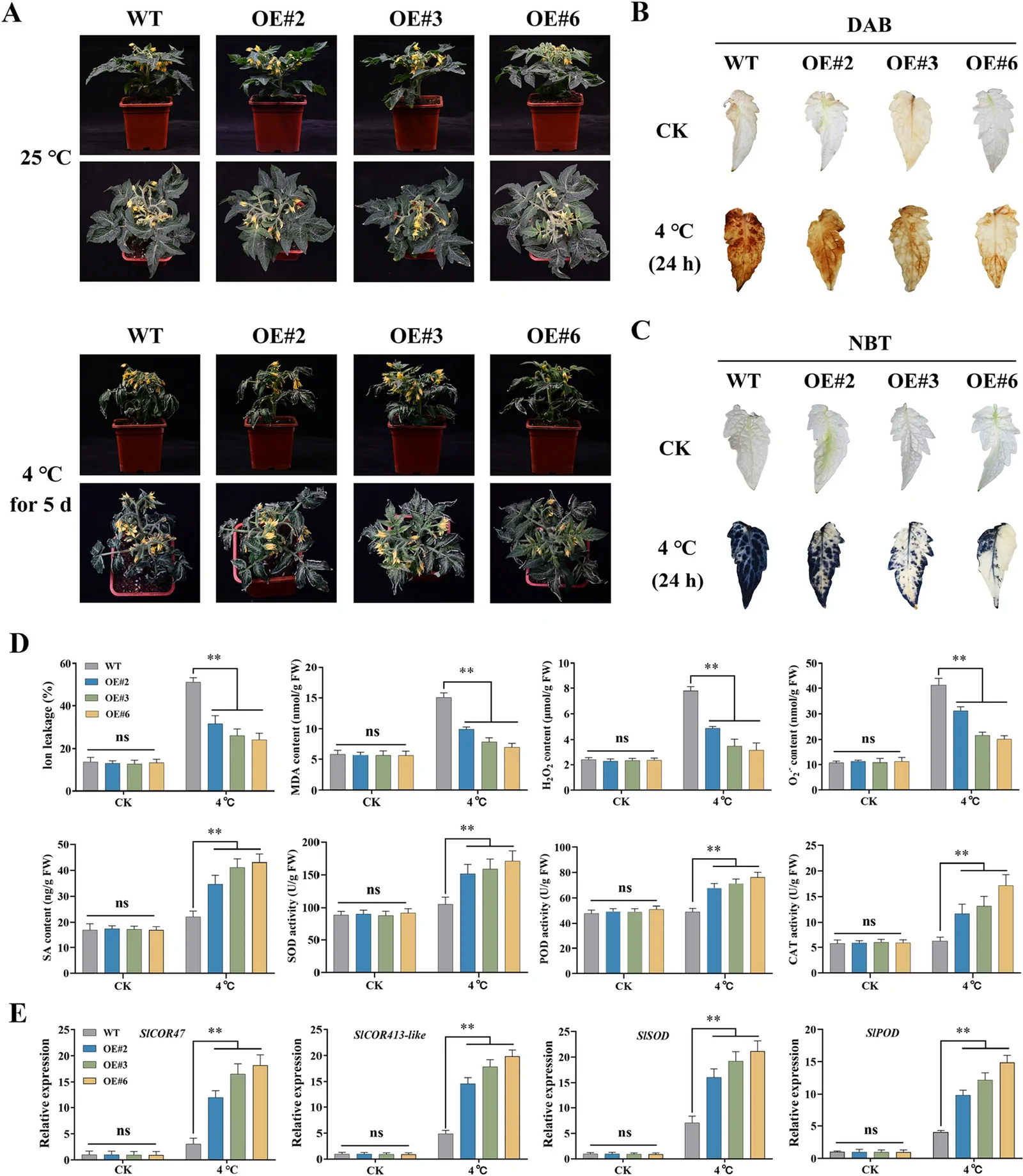
*HuSABP2* enhances the cold tolerance of transgenic tomato lines. **A** Phenotypes of transgenic and WT tomato plants after cold treatment. **B**-**C** DAB and NBT staining. **D**-**E** Changes of ion leakage, MDA, ROS, SA, SOD, POD and CAT in WT and transgenic tomato plants under cold treatment. **E** Expression levels of *SlCORs* in WT and transgenic tomato plants under cold treatment. Error bars indicate the SD of three biological replicates. Significant differences were determined using Student’s *t*-test by comparison with CK (** *p* < 0.01). ns stands for no significant difference

*HuSABP2* was overexpressed in tomato plants to further explore its function. Three T_2_ transgenic lines (OE#2, OE#3, and OE#6) with higher expression levels of *HuSABP2* were selected for analyses. After a 5 d cold treatment at 4 ℃, most leaves of WT plants exhibited obviously wilting symptoms, while the transgenic tomato plants with *HuSABP2* maintained normal growth, with only a few leaves displaying wilting symptoms (Fig. 9A). To investigate the levels of H_2_O_2_ and O_2_^.−^ accumulation, DAB and NBT staining were conducted following a 24 h exposure to 4 ℃. The staining patterns of transgenic and WT plant leaves did not change obviously under normal circumstances. After cold treatment, DAB and NBT staining revealed extensive dark brown and dark blue patches on WT plant leaves, respectively. In contrast, transgenic plant leaves exhibited significantly fewer brown and blue spots (Fig. 9B-C), indicating lower ROS levels in transgenic plants after cold treatment. Compared with WT plants, ion leakage levels, MDA and ROS content in transgenic lines were significantly decreased. In contrast, the endogenous SA content and antioxidant enzyme activities were significantly increased and the expressions of *SlCOR47*, *SlCOR413-like*, *SlSOD* and *SlPOD* were dramatically up-regulated (Fig. 9D-E). These results suggested that *HuSABP2* plays a positive regulatory role in the cold tolerance of transgenic tomato plants.

## Discussion

Cold stress has become a serious environmental risk to agricultural production worldwide, exacerbated by the intensifying effects of global climate change (Kidokoro et al. 2022; Zeng et al. 2025). Exposure to low temperatures has a contrary consequence on plant growth, yield, and geographical distribution, even leading to plant mortality in severe cases (Guo et al. 2018; Kang et al. 2025). Pitaya is a tropical fruit that is a particularly sensitive plant to chilling injury from extreme low-temperature events, which has become a major constraint to the ecological development of the pitaya industry. Understanding the molecular mechanisms behind plant responses to cold stress and developing cold-tolerant cultivars through genetic enhancement are essential for maintaining agricultural productivity, stability, and food security (Zhu 2016). The bZIP TF is pivotal in enhancing tolerance to various abiotic stresses, including cold, drought, and salinity (Yang et al. 2025). VunHY5 and VunMED2 synergistically bind to the G-box/ABRE motif to activate the expression of *VunERD14*, which positively responds to the freezing resistance of plants by reducing membrane lipid peroxidation and improving antioxidant capacity in asparagus bean (*Vigna unguiculata* subsp. *sesquipedalis*) (Liang et al. 2025). In rice (*Oryza sativa*), multiple *bZIP* genes are involved in the cold response process. Notably, a functional polymorphism in *OsbZIP73* determines the difference in cold tolerance between japonica and indica cultivars. Furthermore, OsbZIP73 interacts with OsbZIP71 and enhances cold tolerance by regulating the ABA biosynthesis and ROS homeostasis (Liu et al. 2018a, b). The regulatory functions of bZIP in pitaya remain to be fully elucidated. In this study, a novel cold-responsive HubZIP6 in pitaya was identified as a nuclear protein with transcriptional activation activity (Fig. 1). *HubZIP6* significantly enhances cold tolerance in transgenic *A. thaliana* and tomato plants. The overexpression of *HubZIP6* caused a decrease in ion leakage, MDA content, and ROS accumulation while increased the expression levels of the downstream *COR* genes in transgenic plants, indicating its crucial role in enhancing plant cold tolerance (Figs. 2 and 3).

In recent years, great progress has been made in the cold signaling pathways facilitated by CBF TFs (Ding and Yang 2022). Under cold conditions, CBF TFs are quickly induced and then trigger the expression of *COR* genes, which increase plant tolerance to cold stress (Ding et al. 2024). Several bZIP TFs have been shown to modulate the expression of *CBF*, which can promote or suppress cold tolerance in a variety of plant species. Overexpression of *TabZIP6,* a wheat bZIP TF, reduced the cold tolerance of transgenic seedlings. TabZIP6 may function as a negative regulator in the cold stress response by binding to the promoters of *CBFs*, thereby decreasing the expression of downstream *COR* genes in transgenic *Arabidopsis* seedlings (Cai et al. 2018). MdHY5, an apple bZIP TF, positively regulates the transcription of *MdCBF1* by binding to the G-box motif in the promoter of *MdCBF1*, thereby enhancing the cold tolerance of apple calli and *Arabidopsis* (An et al. 2017). In transgenic plants with overexpression of *HubZIP6,* we found a strong upregulation of *AtCBF1*, *AtCBF2*, *AtCBF3*, *SlCBF1*, *SlCBF2* and *SlCBF3* (Figs. 2 and 3). Similar results have also been reported in pepper, where *CaMYB80* may enhance plant cold tolerance by regulating the expression of *CBF* network genes (Xiao et al. 2024b). This finding suggests that *HubZIP6* may increase cold tolerance through the CBF-COR signaling cascade. Results from DLR, Y1H, and EMSA showed that HubZIP6 directly interacted with the promoters of *HuCBF1* and *HuCBF3*, leading to an enhancement in their transcriptional levels, however, HubZIP6 exhibits no binding activity toward the promoter of *HuCBF2*, suggesting that the interaction between HubZIP6 and *CBF* genes in pitaya is specific (Fig. 4). Those results indicated that HubZIP6 plays a positive regulatory role in cold tolerance through the CBF-dependent pathway.

**Fig. 10:**
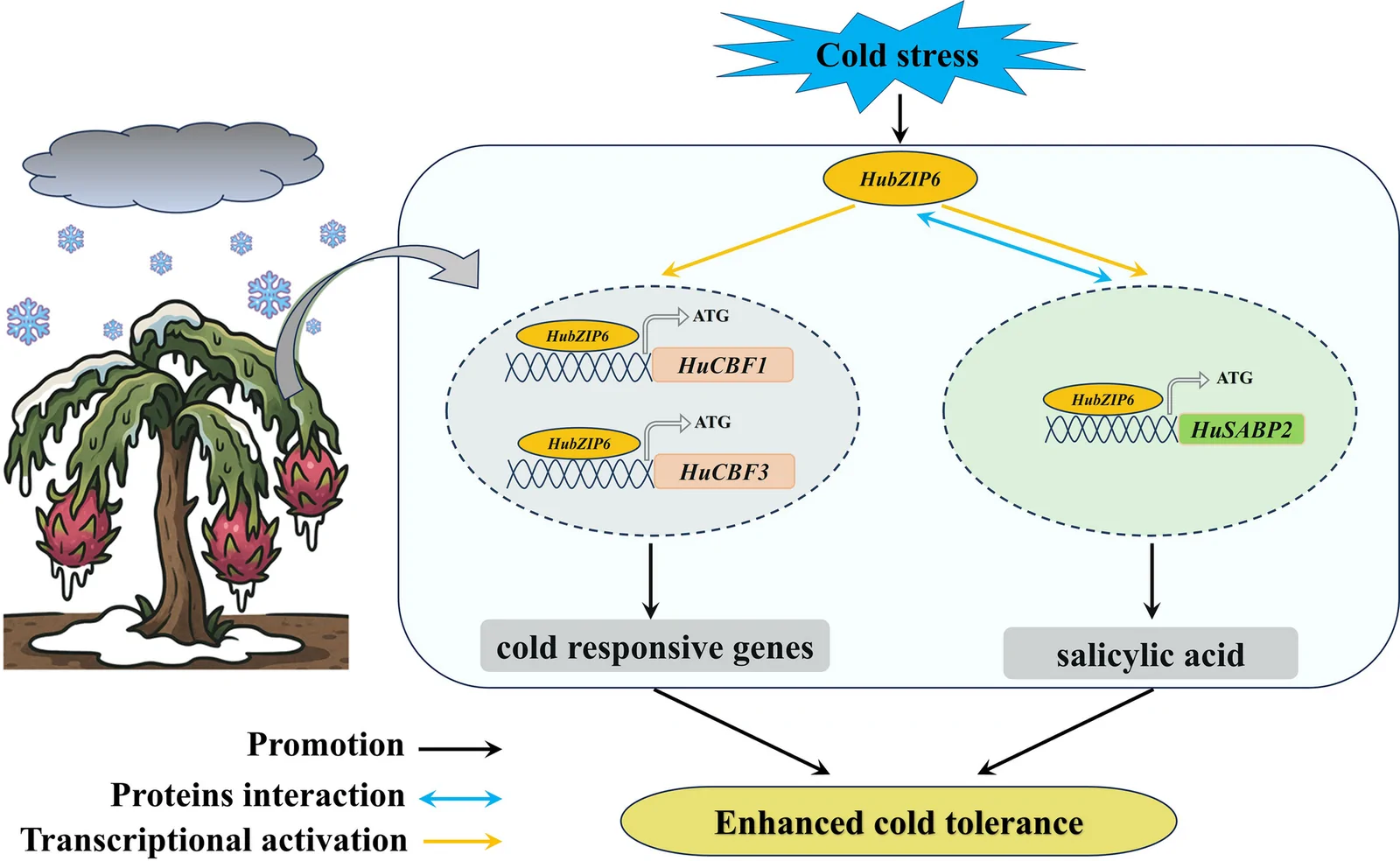
The proposed model of HubZIP6-mediated cold stress-responsive signaling in pitaya. *HubZIP6* is rapidly and significantly induced by cold stress. HubZIP6 promotes the expression of *HuCBF1* and *HuCBF3* by directly binding to their promoters, thereby enhancing plant cold tolerance. HuSABP2 physically interacted with HubZIP6 to synergistically increase the activation of *HuCBF1* and *HuCBF3*. Moreover, HubZIP6 enhances plant cold tolerance by directly targeting the *HuSABP2* gene and activating its transcription, which increases the SA content and reduces the level of ROS in plants

Plant hormones are essential in mediating responses to abiotic stress. ABA, ethylene, JA, and SA, serve as crucial secondary messengers that regulate plant growth, development, morphogenesis, and stress adaptation (Ming et al. 2021; Wang et al. 2024; Yao et al. 2020; Zhang et al. 2025a, b). Among these, SA is of particular importance in mediating defense signaling, secondary metabolism, and responses to abiotic stresses (Wildermuth et al. 2002; Zhu et al. 2025). In citrus, the regulatory module composed of CtrTGA2-CtrP5CS1 functions in a SA-dependent manner, and confers cold tolerance to plants by regulating proline synthesis (Xiao et al. 2024c). SA-binding proteins (SABPs) play a vital part in regulating SA-mediated defense (Pokotylo et al. 2022). In particular, SABP2 is an esterase that has both antioxidant enzyme and MeSA hydrolase activity (Khokon et al. 2011). Under stress conditions, SABP2 catalyzes inactive MeSA into active SA, leading to the enhancement of stress resistance (Wildermuth et al. 2002). In potato, results from virus-mediated gene silencing of *StSABP2* and transient expression of *StSABP2* in tobacco showed that StSABP2 positively regulates plant resistance to *Phytophthora infestans*, and this process is achieved by mediating the transcription of SA signaling and defense-related genes. Furthermore, StNAC2, as an upstream regulator of *StSABP2*, could activate its transcription. The StNAC2-StSABP2 regulatory module regulates potato resistance to *P. infestans* by positively mediating the SA pathway (Yan et al. 2025). Overexpression of the *SABP2* could enhance the tolerance of transgenic sweet oranges to Huanglongbing (HLB, also known as citrus greening disease) (Soares et al. 2022). Despite its importance, the role of *SABP2* in the cold stress response of pitaya has not been reported. In the current study, a cold-responsive HuSABP2 protein was identified (Fig. 7), which significantly enriched cold tolerance by increasing SA synthesis, boosting the activity of antioxidant enzymes, and reducing the accumulation of ROS when *HuSABP2* was expressed in *Arabidopsis* and tomato plants (Figs. 8 and 9). HuSABP2 physically interacted with HubZIP6 to synergistically amplify the activation of *HuCBF1* and *HuCBF3* (Fig. 5)*.* Furthermore, HubZIP6 can directly target the *HuSABP2* gene and activate its transcription while HuCBF1 and HuCBF3 show no regulatory effect on the transcription of *HuSABP2* (Fig. 6 and Figure S6). These findings indicate a cooperative action of HubZIP6 and HuSABP2 in regulating cold tolerance in pitaya (Fig. 10). In summary, the study provides novel insights into the regulatory mechanisms of the bZIP TFs govern pitaya tolerance to cold stress. *HubZIP6* and *HuSABP2* revealed in this study provide important gene resources to enable the genetic enhancement of cold tolerance in pitaya and to facilitate cold tolerance breeding in related plants.

## Materials and methods

### Plant materials and growth conditions

The cuttings of two pitaya cultivars, i.e., ‘SCAU-NH’ (resistant to low temperature) and ‘SCAU-KX’ (sensitive to low temperature), were used as materials for gene cloning and qRT-PCR. Pitaya were planted in an orchard from South China Agricultural University, Guangzhou, China. Tobacco (*Nicotiana benthamiana*) was used for the functional verification assays. The *Arabidopsis* ecotype Columbia-0 and tomato (Micro-tom) were used for cultivating transgenic plants. The *bzip6* and *sabp2* mutant *Arabidopsis* lines (SALK_077086C and SALK_204268C) were obtained from Arashare (https://www.arashare.cn/index). Tobacco, *Arabidopsis*, and tomato seedlings were grown in a climate chamber at the following conditions: diurnal temperature cycle 22/18 ℃, 16 h light/8 h dark light cycle, and 60–70% relative humidity.

### Gene cloning and expression analyses

In accordance with the instructions provided by the manufacturer, the EASYspin Plus Complex Plant RNA Extraction Kit (Aidlab Biotechnology, Beijing, China) was used to extract the total RNA from samples of pitaya. Following that, the RNA was collected and reversely-transcribed into single-stranded complementary DNA (cDNA) according to the PrimeScript™ RT Reagent Kit with gDNA Eraser (TaKaRa, Japan). TBtools software was used to design and verify the specificity of primer sequences for quantitative real-time PCR (qRT-PCR) (Chen et al. 2020). The *HuActin(1)* gene was used as the internal control (Chen et al. 2019). qRT-PCR experiments was perform on a CFX384 Real-Time PCR Detection System (C1000 Touch Thermal Cycler, Bio-Rad, California, USA). The 2^−∆∆C^T method was used to calculate the gene expression (Livak and Schmittgen 2001). Three biological replicates were performed. The full-length coding sequences (CDS) of *HubZIP6* and *HuSABP2* were cloned using the ClonExpress Ultra One Step Cloning Kit V3 (Vazyme, Nanjing, China). The primer sequences were listed in Supplementary File S2.

### Subcellular localization analyses

The CDS of *HubZIP6* and *HuSABP2* without the stop codon were inserted into the pGreen-35S-GFP vector to create C-terminal fusions with the green fluorescent protein (GFP). The primers are listed in Supplementary File S2. Following this, the constructs pGreen-HubZIP6/HuSABP2-35S-GFP and a vector control were introduced into *Agrobacterium tumefaciens* strain GV3101 (pSoup-p19) and applied to the abaxial leaf surface of the *N. benthamiana* leaves. Leaf samples underwent incubation under standard growth conditions for a duration of 2 d and then they were collected for analysis and GFP assessment. Fluorescence was detected under a fluorescence microscope (ZEISS LCM-800, Germany).

### Analysis of yeast cell transcriptional activation

The CDS of *HubZIP6* was inserted into the pGBKT7 vector, which includes the GAL4 domain. Y2H Gold yeast competent cells (Coolaber, China) were separately transformed with the positive control (pGBKT7-p53), the negative control (pGBKT7-Lam), and the pGBKT7-HubZIP6 construct. The primers are provided in Supplementary File S2. Transformed yeast cells were then cultivated on SD/-Trp and SD/-Trp-His-Ade medium for a duration of 3 to 5 d. Following incubation with 20 µg/ml X-α-galactosidase (X-α-Gal) for 10–30 min, the proliferation of yeast cells and the enzymatic activity of X-α-Gal were assessed.

### Verification of cold-responsive genes by a yeast system

The fragments of *HubZIP6* and *HuSABP2* were cloned into pYES2 using ClonExpress Ultra One Step Cloning Kit v3 (Vazyme, Nanjing). Primers are given in Supplementary File S2. Empty vectors and plasmids with pYES2-HubZIP6 and pYES2-HuSABP2 were respectively transformed into a BY4741 yeast strain (Weidi, China) and were screened on SD/-Ura medium. Positive yeast clones were suspended in 1 mL of sterile water. Then, 3 μL of the suspension was spotted on SG/-Ura medium for phenotypic verification. The medium was further incubated at 30 ℃ (CK), 25 ℃, 20 ℃, 15 ℃, and 10 ℃ for 5 d. Images were taken after 3–5 d. Three biological replicates were performed for each treatment.

### GUS activity analysis

2000 bp promoters of *HubZIP6* and *HuSABP2* were cloned using the Phanta Max Master Mix (Dye Plus) (Vazyme, Nanjing). Thereafter, the promoters were ligated together in the pCAMBIA1391-GFP construct, and the recombinant plasmids were introduced into the *A. tumefaciens* strain GV3101 (pSoup-p19) and subsequently injected into the abaxial surface of the *N. benthamiana* leaves. Infected leaf tissues were harvested for GUS staining after incubating them in the controlled growth environment for 2 d. GUS stain and GUS enzyme activity were performed using the GUS Stain Kit manual (Coolaber, China).

### *Arabidopsis* and tomato transformation

*HubZIP6* and *HuSABP2* CDS were placed into the pPZP6K90 plasmid driven by the 35S promoter and introduced into *A. tumefaciens* strain GV3101. Wild-type *Arabidopsis* (Col-0) were then transferred to recombinant plasmids with the aid of the floral dip technique. Positive *Arabidopsis* plantlets were selected on 1/2 MS medium with 100 mg/L kanamycin (Kan), and their presence was confirmed by PCR analyses. qRT-PCR was used to ascertain the expression levels of *HubZIP6* and *HuSABP2* in each transgenic lines (primer details were shown in Supplementary File S2). Three homozygous T_3_ transgenic lines were used for further analysis. In the case of tomato plants, 9–10-day-old cotyledons were used as explants for *Agrobacterium*-mediated transformation. Transformed cotyledons were then cultured in MS solid medium supplemented with 2.0 mg/L 6-BA +0.2 mg/L IAA +50 mg/L Kan to stimulate adventitious shoot induction. PCR was used to verify the positive lines, and qRT-PCR was used to quantify the expression levels of *HubZIP6*. After several sets of screening, two homozygous T_2_ transgenic lines were used for further studies.

### Cold stress tolerance assay

One-week old wild-type (WT) plants and transgenic *Arabidopsis* lines were acclimated for 48 h at 4 ℃ in darkness, followed by exposing them to −6 ℃ over a period of 6 h and incubating them at 4 ℃ in the dark over a period of 12 h. The plants were then moved to the normal conditions (22 ℃), where they were left to recover for 6 d. Before the treatment and following the recovery period, phenotypic changes were recorded. *HubZIP6*-transgenic and WT tomato plants were similarly subjected to cold treatment. Experimental conditions were as follows: (i) the control group, which was kept in normal temperature conditions; and (ii) it was exposed to 4 ℃ for 6 d. Phenotypic variations were noted, and leaf samples were harvested and immediately frozen at −80 ℃ in liquid nitrogen until further analysis. After 24 h of treatment at 4 ℃, the samples were collected for DAB and NBT analyses. The processes of staining were performed as per the manufacturer’s guidelines of the DAB and NBT kits (Coolaber, China). All experiments were repeated three times.

### Determination of physiological and biochemical indicators

The contents of ion leakage, ROS, malondialdehyde (MDA), proline, superoxide dismutase (SOD), peroxidase (POD), and catalase (‌CAT) activities were measured following the previous studies (Xiao et al. 2024a). The SA extraction and determination were performed as described by (Pan et al. 2010) and (Zhang et al. 2015). The ion leakage assay consisted of three independent experiments and each experiment contained five biological replicates. Samples were washed and placed in centrifuge tubes with deionized water. Ion leakage was measured initially (S_1_) after shaking for 30 min, and then boiling for 15 min and shaking for 1 h (S_2_). Blank control ion leakage was recorded as S_0_. Ion leakage was calculated using the following formula: ion leakage = (S_1_—S_0_)/(S_2_—S_0_).

### Dual-luciferase reporter assays in *N. benthamiana* leaves

Leaves of *N. benthamiana* were used to assess the transcriptional activity of HubZIP6. The full-length CDS of *HubZIP6* was cloned into the pGreen II BD-62-SK vector to produce the effector construct. The pGreen II 0800-LUC vector containing the minimal CaMV 35S TATA box fused with five tandem GAL4 DNA-binding sites (5 × GAL4) was used as reporter. *A. tumefaciens* strain GV3101 (pSoup-p19) was respectively transformed with pGreen II BD-62-SK-HubZIP6, pGreen II BD-62-SK (negative control), pGreen II BD-62-SK-VP16 (positive control), and the reporter plasmid pGreen II 0800-LUC. A respective mixture of the effector and reporter strains (9:1) was infiltrated into *N. benthamiana* leaves. Luciferase activity was assessed after 3 d post-infiltration using the dual-luciferase assay kit (Promega, USA), and LUC/REN ratio was calculated. The primer sequences are given in supplementary File S2. Promoters of three *COR* genes (*HuCBF1*, *HuCBF2* and *HuCBF3*) were analyzed based on the Plant CARE database (http://bioinformatics.psb.ugent.be/webtools/plantcare/html/). The results are provided in Supplementary Tables. For functional validation, the CDS of *HubZIP6* were cloned into the pGreen II 62-SK vector as effectors and 2000 bp promoter fragments of each target gene were cloned into the pGreen II 0800-LUC vector as reporters. Both the effector and reporter constructs were individually transformed into *A. tumefaciens* GV3101 (pSoup-p19) and then were co-infiltrated into *N. benthamiana* leaves at a ratio of 9:1. After 48 h, the LUC fluorescence was carried out and LUC/REN were determined.

### Y1H and EMSA experiments

*HubZIP6* was inserted in the pGADT7, and the full-length and the fragments of *HuCBF1*, *HuCBF3*, and *HuSABP2* promoters with ACGT-motif were inserted in the pAbAi fragment for yeast one-hybrid (Y1H). The primers were listed in Supplementary File S2. Prey and bait constructs, positive control (pAbAi-p53 + pGADT7-p53), and negative control (pAbAi-p53 + pGADT7) were separately transferred into yeast Y1HGold. After 3–5 d of incubation at 30 ℃ in the dark, each group’s growth was evaluated to determine the interaction that existed between the promoters of *HuCBF1, HuCBF3, HuSABP2*, and *HuZIP6*. The *HubZIP6* CDS were inserted into the MBP-tag fusion pMAL-c2X construct to allow for expression and purification of the MBP-HubZIP6 protein of the BL21 (DE3) cells. The probes were ordered as custom-synthesized biotin-tagged *HuCBF1*, *HuCBF3*, and *HuSABP2*-specific probes (the primer sequences are described in Supplementary File S2). Electrophoretic mobility shift assay (EMSA) was performed in line with the guidelines outlined in the EMSA/Gel-Shift kit manual, which was supplied by Beyotime Biotechnology (Shanghai, China). The primer sequences of EMSA were described in Supplementary File S2.

### Y2H and luciferase complementation imaging (LCI) assays

The procedure of library screening was carried out as follows: the full-length CDS of *HubZIP6* was cloned into the pGBKT7 vector and then transferred to the yeast strain Y2HGold. The yeast cells were used to inoculate the introduction of the pitaya cDNA library, and then were cultured on the selective medium SD-Leu/Trp/Ade/His (LWAH). The entire CDS of *HuSABP2* was cloned into the pGADT7 to validate the interaction, followed by co-transformation into the Y2H strain together with BD-HubZIP6. The primers are listed in Supplementary File S2. The transformed cells were grown on selective media SD-Leu/Trp (LW) and SD-LWAH. The whole CDS of *HubZIP6* and *HuSABP2* without the termination codon were cloned into the pCAMBIA1300-nLUC (nLUC) and pCAMBIA1300-cLUC (cLUC) plasmids, respectively (Supplementary File S2). The recombinant plasmids and P19 auxiliary plasmid were transferred into the *A. tumefaciens* GV3101 strain (Weidi, China) and then injected abaxial surface of the *N. benthamiana* leaves. The abaxial surfaces of the *N. benthamiana* leaves, which were the same areas on which the bacterial solution had previously been sprayed, were treated with D-luciferin potassium salt at a concentration of 0.1 mM. This treatment was carried out after an incubation period of 48 h at 22 ℃ under long-day circumstances (a cycle 16 h of light and 8 h of darkness). The *N. benthamiana* leaves were then harvested, and fluorescence was detected under the fluorescence chemiluminescence gel imaging system (Berthold Technologies, Germany).

### Pull-down assay

The CDS of *HuSABP2* was amplified and integrated into the pGEX-6P-1-GST vector (GE Healthcare, USA) to generate the GST-HuSABP2 fusion protein. Both GST-HuSABP2 and MBP-HubZIP6 (same as that of EMSA) were expressed in Rosetta (DE3) cells (TransGen Biotech, China) and subsequently purified using GST-tag resin (Beyotime, Shanghai, China) and MBP-tag agarose (Qiagen, Hilden, Germany) following the manufacturer’s manual. For the pull-down assay, GST and GST-HuSABP2 attached to the GST-tag resin were incubated with MBP-HubZIP6 in GST pull-down binding buffer (50 mM Tris–HCl, 200 mM NaCl, 1 mM EDTA, 1% NP-40, 1 mM DTT and 10 mM MgCl_2_, pH 8.0). After 4 h of gentle rotation at 4 ℃, the mixture was washed with GST pull-down washing buffer (50 mM Tris–HCl, 50 mM GSH, 400 mM NaCl, 1 mM EDTA and 1 mM DTT, pH 8.0). The eluted proteins were then analyzed using immunoblotting with anti-GST and anti-MBP antibodies (Beyotime, Shanghai, China).

### Statistical analysis

The data, represented as means ± standard deviation (SD) from three replicates. Statistical differences were calculated using a two-sided Student’s *t*-test in SPSS software (IBM, SPSS 22) and Graphpad Prism v.10.0. *p < 0.05, and **p < 0.01 denoted different statistical significance. ns stands for no significant difference.

## Supplementary Information

Additional file 1: Supplementary Figure S1. Expression analyse of the bZIP gene family in pitaya under cold stress. (A) Transcription levels of the bZIP family in two pitaya varieties under cold conditions (4 ℃). (B) Relative expression levels of differential genes in two pitaya varieties under cold conditions (4 ℃). Error bars indicate the SD of three biological replicates. Supplementary Figure S2. Schematic diagrams of the chromosomal location of the HubZIP genes. Supplementary Figure S3. Identification and expression analyses of *HubZIP6* in transgenic Arabidopsis lines. (A) Identification of transformed Arabidopsis lines by PCR. M: DNA marker 2000; A1-A13: Arabidopsis plants transformed with *HubZIP6*; WT: wild type; P1: positive control (pPZP6K90-HubZIP6). (B) Expression levels of *HubZIP6* in WT and transgenic plants. Error bars indicate the SD of three biological replicates. Supplementary Figure S4. *HubZIP6* mutant lines exhibit decreased cold tolerance in *Arabidopsis*. (A) Phenotypic comparison of 4-week-old mutant and WT *Arabidopsis* after cold treatment. (B-C) Changes of ion leakage and MDA content in mutant and WT *Arabidopsis* after cold treatment. (D) Expression levels of *AtCBFs* and *AtCOR47* in mutant and WT *Arabidopsis* under cold stress. Error bars indicate the SD of three biological replicates. Significant differences were determined using Student’s *t*-test by comparison to CK (** *p* < 0.01). ns stands for no significant difference. Supplementary Figure S5. Identification and expression analyses of *HubZIP6* in transgenic tomato lines. (A) Identification of transformed tomato lines by PCR. M: DNA marker 2000; A1-A11: tomato plants transformed with the *HubZIP6*; WT: wide type (negative control); P1: positive control (pPZP6K90-HubZIP6). (B) Expression levels of *HubZIP6* in WT and transgenic plants. Error bars indicate the SD of three biological replicates. Supplementary Figure S6. DLR analysis of the activation between HuCBF1, HuCBF3 and the promoter of *HuSABP2*. Error bars indicate the SD of three biological replicates and ns stands for no significant difference (One-way ANOVA, Tukey’s test, *p* < 0.05). Supplementary Figure S7. *HuSABP2* mutant lines exhibit decreased cold tolerance in *Arabidopsis*. (A) Phenotypic comparison of 4-week-old mutant and WT *Arabidopsis* after cold treatment. (B-C) Changes of ion leakage and SA content in mutant and WT *Arabidopsis* after cold treatment. (D) Expression levels of *AtSOD*, *AtPOD*, *AtCAT*, and *AtICS1* in mutant and WT *Arabidopsis* under cold stress. Error bars indicate the SD of three biological replicates in all graphs. Significant differences were determined using Student’s *t*-test by comparison to CK (** *p* < 0.01). ns stands for no significant difference.

Additional file 2. The sequences of HubZIP6 and HuSABP2.
Additional file 3: Supplementary Table 1. The list of primer pairs used in this study.

Additional file 4: Supplementary Table 2. Cis-element analysis of the *HuCBF* promoters.

## Data Availability

RNA-Seq data from four points (0, 3, 6, and 12 h) of ‘SCAU-NH’ and ‘SCAU-KX’ pitayas after cold treatment have been deposited in the NCBI SRA database under accession number PRJNA1335801.

## Abbreviations

bZIP: Basic leucine zipper
CBF: C-repeat-binding factor
COR: Cold-regulated
DLR: Dual-Luciferase reporter assay
GUS: β-Glucuronidase
ROS: Reactive oxygen
SA: Salicylic acid
Y1H: Yeast one hybrid
Y2H: Yeast two hybrid
