# Supplementary material for: Enhancing cold tolerance through HubZIP6-mediated CBF activation and salicylic acid signaling in pitaya
Source: Mol Hortic. 2026 Sep 3;6:64. doi: 10.1186/s43897-026-00258-3 (PMC13540873; doi:10.1186/s43897-026-00258-3)
Supplement: Supplementary file 1 [file 43897_2026_258_MOESM1_ESM.docx]

**Supplementary Figure. S1** Expression analyse of the *bZIP* gene family in pitaya under cold stress. (A) Transcription levels of the *bZIP* family in two pitaya varieties under cold conditions (4 ℃). (B) Relative expression levels of differential genes in two pitaya varieties under cold conditions (4 ℃). Error bars indicate the SD of three biological replicates.

**Supplementary Figure. S2** Schematic diagrams of the chromosomal location of the *HubZIP* genes.

**Supplementary Figure. S3** Identification and expression analyses of *HubZIP6* in transgenic Arabidopsis lines. (A) Identification of transformed Arabidopsis lines by PCR. M: DNA marker 2000; A1-A13: Arabidopsis plants transformed with *HubZIP6*; WT: wild type; P1: positive control (pPZP6K90-HubZIP6). (B) Expression levels of *HubZIP6* in WT and transgenic plants. Error bars indicate the SD of three biological replicates.

**Supplementary****Figure. S4** *HubZIP6* mutant lines exhibit decreased cold tolerance in *Arabidopsis*. (A) Phenotypic comparison of 4-week-old mutant and WT *Arabidopsis* after cold treatment. (B-C) Changes of ion leakage and MDA content in mutant and WT *Arabidopsis* after cold treatment. (D) Expression levels of *AtCBFs* and *AtCOR47* in mutant and WT *Arabidopsis* under cold stress. Error bars indicate the SD of three biological replicates. Significant differences were determined using Student’s *t*-test by comparison to CK (** *p* < 0.01). ns stands for no significant difference.

**Supplementary****Figure. S5** Identification and expression analyses of *HubZIP6* in transgenic tomato lines. (A) Identification of transformed tomato lines by PCR. M: DNA marker 2000; A1-A11: tomato plants transformed with the *HubZIP6*; WT: wide type (negative control); P1: positive control (pPZP6K90-HubZIP6). (B) Expression levels of *HubZIP6* in WT and transgenic plants. Error bars indicate the SD of three biological replicates.

**Supplementary Figure. S6** DLR analysis of the activation between HuCBF1, HuCBF3 and the promoter of *HuSABP2*. Error bars indicate the SD of three biological replicates and ns stands for no significant difference (One-way ANOVA, Tukey's test, *p* < 0.05).

**Supplementary Figure. S7** *HuSABP2* mutant lines exhibit decreased cold tolerance in *Arabidopsis*. (A) Phenotypic comparison of 4-week-old mutant and WT *Arabidopsis* after cold treatment. (B-C) Changes of ion leakage and SA content in mutant and WT *Arabidopsis* after cold treatment. (D) Expression levels of *AtSOD*, *AtPOD*, *AtCAT*, and *AtICS1* in mutant and WT *Arabidopsis* under cold stress. Error bars indicate the SD of three biological replicates in all graphs. Significant differences were determined using Student’s *t*-test by comparison to CK (** *p* < 0.01). ns stands for no significant difference.
