# Supplementary material for: Enhancing cold tolerance through HubZIP6-mediated CBF activation and salicylic acid signaling in pitaya
Source: Mol Hortic. 2026 Sep 3;6:64. doi: 10.1186/s43897-026-00258-3 (PMC13540873; doi:10.1186/s43897-026-00258-3)
Supplement: Supplementary file 2 [file 43897_2026_258_MOESM2_ESM.docx]

CDS sequences

**>HubZIP6**

ATGATGCAGGTGAGTTCAGGGTCAGAATCGGACCCTAGATACGCCAACATGGACGACAAGAAGCGCAAGCGGATGATTTCGAACCGCGAGTCGGCTAGGCGATCGAGGATGAAGAAGCAGCAGCATATGGACCAGCTGCTGAACCAGGTGACCACGCTGAAATCAGAGATTGCTCAACATTCTCAGACAATCGATGCCGTCTCGCAGCGCTACGTTGCAGTTCAATCGGAGAACAATGTGCTTCGAGCCAAGCTGATGGAGCTCACTGATAGGCTCACATCCCTGAATTCCGTGCTCAAGATGGTGGAAGAGGTTGGCGGCTTCTCAATGGATATTCCTGATGTTCTGCTGGAGCCATGGCACTTGCCGTGCGCTCTTCAACCAGTATCCAACATCTTCCAATGCTAA

**>HuSABP2**

ATGGAAGATGAGGGGAAGAAATTAGGAGGGAAGCATTATGTGCTGGTACATGGAGCATGCCATGGAGCATGGTGCTGGTTCAAGGTCAAGCCTCTCCTTGAGGAAGCCGGCCACCGGGTCACGACCCTTGACATGGCGGCTTCCGGCATCCACCCCGCCACCATACACCAACTCCACACTCTCCGCGACTACTGTCGGCCGTTGATTGACTTCATGGGTTCGCTCCCTGATGATCAGAAGGTCATTCTCGTTGGGCATAGCCTTGGAGGACTCAACGTGGCTGTGATCATCGAGATGTTCCCTTACAAAATAGAAGCTGCGGTCTTCCTGGCTGCTTTCCTGCCTGATACGCATCACCACCCCGCATTTGTGGTCGATCAGTTTGCTGCAAGACTGCCAGAAGATTTTTGGCTTGACACTGAGTTTAAGAGCACCGGAGACCCTAACGAGTCCCTCACGACCATGTTGTTCGGCCCCAAGTTCGTGTCCTTGCTCTATCACTTGACCCCTCTTGAGTATCATTTTCTGTTTTCTGGTGTCAAGGATTTTGAGCTTGGTATGATGCTGAAAAGGCCATCGTCGTTCTTCCTGCATGATCTGCGAAAGGCAGACAAGTTTTCAAAGGAGAAGCACGAGTCAGTGAGGCGGGTTTATGTGGCGTGCGAGGAAGATAAAGCAATAACGAAAGATTTCCAGCTCTGGATGGTTGAGAATGGTGGAGTGAAAGAGGTGAAGGAGCTTAAAGGTTCAGACCATATGCCTATGTTAAGCATGCCTCAAAAACTCTCTGATTGCCTCATAGAAATTGCCCGTAAATGTTGA

Protein sequences

**>HubZIP6**

MMQVSSGSESDPRYANMDDKKRKRMISNRESARRSRMKKQQHMDQLLNQVTTLKSEIAQHSQTIDAVSQRYVAVQSENNVLRAKLMELTDRLTSLNSVLKMVEEVGGFSMDIPDVLLEPWHLPCALQPVSNIFQC

**>HuSABP2**

MEDEGKKLGGKHYVLVHGACHGAWCWFKVKPLLEEAGHRVTTLDMAASGIHPATIHQLHTLRDYCRPLIDFMGSLPDDQKVILVGHSLGGLNVAVIIEMFPYKIEAAVFLAAFLPDTHHHPAFVVDQFAARLPEDFWLDTEFKSTGDPNESLTTMLFGPKFVSLLYHLTPLEDFELGMMLKRPSSFFLHDLRKADKFSKEKHESVRRVYVACEEDKAITKDFQLWMVENGGVKEVKELKGSDHMPMLSMPQKLSDCLIEIARKC

Promoter sequence

**>HubZIP6**

TCAACTACAAGTTCGCTACATTCCTACTCAGTCTCAAGTGGCAGACATCTTCATTGAGGGTCTTTCTTCACAGAAGTTCTATCAGTTCAAGGCTAATCTGTTCGTGGTGCCCCCGTTCAGATTGAGGGGATGTAATAGTGTAATATATCTATGTAACATATATTTGTTGGGCCTTGTAATTATCTCGTATCAAACCCTAATTACGAATATAAATATACACCCAGACAACCACATCAGGGCAGGTGGCGTAATTTCTGCACACACCCATTAACACGCCTAATCAATCCCTCCCTTAGGAAAGCTTTTCCTACCCAGCTCCATTAGAAGTGTAGCCTGGGTTATAGCCGAACATTACATCTAGTCACACTTGTAAAATTAGGCCAATAAAATCAATTGCCCCACCGCTTAACTGCAAAAGCATAATTGAACACATCAATTCTCCTAAATTACAAACTCACAACTTATCACATTCAAATCATAAACTCCTTATAAAAACCGATATTTACTACTTTTAAGGTGTAAAAGTTTGAATAGTGATCTTAGCAATGAAAAAGAAAAGAATTGTACCTCATTTGAAGCTTTGAATTGAATTGTAAATGCAATACATTACATGAGAATCAGTATGGTTTACTTAATTTATAATTTAGTAGCTTGAACTTCTTCAAATTAAGTGCAATTTTGATATTGAATAGGTCAATCATGTCACTTTAAAAAATGAACAGAATTTTTTGAGTAATTTTGAACACATTTAATCACAACTATATAGTTAAAGATATGTTACTTTTCAGTTAGCTATTCAAAAAATGAAAATACCTTTTTGATCTAATTTAATACTTTGTTATATTTTGTACAAACTAATTTATGCCTTGTGCCAATCACAAAACAAACGAATCATTTATACTATAAAAAAGGCATTTCTTCAAACTATTCTTATTCTTGGAAAATCTATTTTATGACTTCTTTTAGGCAACAATTAAAGTTTATATGTTACTGAACACAAAAAAAAAATTTATATGTTACTTTGTTCATTCCATTTTTATTTTACTAAACGAATTAAGAAAGCCAAGAAAAATTAACCGTTAGACAATAATACATCATTGATCTGGAGGAAATGGTGCAAGACCTTTTTTTGTTATTTAAAGAAATGATTAGTGTATTATTAGTCACTTGAAAAGTTATTGATCAGTAGATGATCATTGTTCATATTAATAATTGTTTATTTGTTTTTGTCTCTTAATGATTGATATATGAATTTCTTTATGTACTAATTTTATTTGGAAAAAAATTATGACATATCATTCAATATGGATTTTGAAAAAAAGTCAGCAAATCTTTATTAATCCTAAAACTTATCCAAAACTTGTTAGCCGAAAAGGGGGAAAAGAAACTTATCCAAATCTTTATGTGGGAGAATAGAAAAGTAAATCTGGAAATCCTGGAATAATAGGAAACAATGTCGAAGACCGGGGGAGTCGCAATCGTTGACTAAGAGTCGGAGGGTCAAAAACGTCAATACGCGTACAATGTCCAACCTGTCGTCGCAGGGCATTCTCATTGCCAGCTTAAAGCTTTCCTTGTTCTGTTTTCCAATCCGGACCACCCTTTTCTCCGCTCTTTTCCGGGCATCTGTTTTCCTATAAAACCATCGCCCACAACACACCTTCCAATCTTGATTTTCCATCCTGTTTGTCCCTGGCTGCCTTTCTCATCTTCTCTCCTCTTGATTTCTTCTTCCCCTAATCTTTCTTCAAATTTGTTTGTTATTATTGCATGAATTGCGACTCGATGTTTATATGAGCTATCAGGTCCAGATGCGTCGAATTCGAATTCTCCATTCTTTCTCGGTTGTTTTTCTCTACTGGTTCTACGTCTTTTCATTGATCAATTAGTTCATTGATTCCCCGCCTTAGTTAGAGTACCTGACATCCATCTCTTCGATTTATAATCCAATATAGTTAACCCTTAGCAGCAGCTCTTAGTTTAGATCGAGGGAAAGAACG

**>HuSABP2**

GCCAAGATATAGGGGACCTTTATTTACATGAGAGTTTCACAAATAAAGCCCCATTTAAGGCGATCGTATGCCTTTTTCAAGTAAATCTTTATCGTCATGGTTCCTCTGGTTCCTTTATTTGTACGCATAGTATGGAGAATCTCTTGCATGACAACAACATTATCACCAACTTGACGACTTTGGACAAAGCTGGTTTCGGTCGGGCAATTAAATCAGGTTAGATCAACTTCAGTCTTTGCACTATGGCTTTGGGAGCGATCTTATAAATCATATTACACAATCCATCACAAACTGTGGGTTCTACACTTTAGGGATAGGAGCAAGATAAGTATCATCAAAAGTTAAAACCACATGGCATCTGGGCACCACGGAACACATCGAGGACCCCCTCACAAATAGGGTTGCTAACAAGGGACAATTGCTGTTTTTTGTGGTGGGGGGGGGGGGGGGCGTACAGAAGAGGGGTTCCTTGACTTCATCAGAAGAAAAAGGTTTGGCGAAAGCAGCAATTTGATCATATTTTAACAGAGGGAAATTTTATTAATTGGCAATAAATTTGGACTACATTGCTCAACTATATTATTTGTATACAAGAGTCATGAATCTCACTTGTAGCTGAATCAAAAAATGTTGATTTGCTTATGAGGTTGGACACCACGCACCATGTCTCCATCTCTTTGCACTCAATGTTAGAATCCAATCCGCTATCACCTACATTATTTTCTTTATTTTTTCCAAGTTGTTTCCTTATTAAATATTGTACAATGCAATTGGATTGATATTAAACTAGACCTAAAAAGAAAAAATGAGACAAGCTCAGCTAAAATTTGGTAGGCCTTTACAGTAAAGCACGGGAGGGAGAAGTTCAAGAATTTTAACAAAGTTTATTTCACTATTTTACCTGTTTAAAATGACTCTTTATAACATAGGTTGTACTTTTTATTTTTTTTTTTAAAATAGTTAGTACTTAGGCCAGTTGATGGAGAGACGTCCAATGTTTATTTAGGCTATTTTAAAATGTCTACATATACTATTCTTAACTTTTTATAAACAGTATATTATAATTTTTTGGAGAGCCAGGGTGTCCATGGACACCCCTGAACTCTACAAAGATCTGCACATGGCTTGGAGAGAAATGTTTCCCGTGGTCCTCTTTTCAGCCATCAGTCGCTTTTACAGAACGTACAAATATTACACACCATAAGAACTTTACAACTATAAGTCATCTAAACAATTTCTGACTTTACAAGTATAATTCATTTAGAATACAATCTCTGGCTAGATTCCTAATATTTACTAAGGGAACCTCTTCTCAAGATTTTTTTTTTAAATAGATACTTAATTTAGCTAGAAAATTGAAAAAATAAAATAATTGAAACTGCATAAGCTGTAAATTTACTTAGTATAACGGAAGAACACTGATGTCTGGGGTGATGGTTTTTTTTTTAATAGTCATAAATTTAATTACGTTAGTTTATTAGACATTAAACATGGATTAAATTAGACACCTAGAAAATAAATATACAAAAAAATCTCCCATCAGTTACAAATTAGGTTATCTTCTTAATTAATTTGTTGCAAATATTTTCTGGAAGTGCTAGCATATGTAATAGATCAGAATAAAACAAATCATACAAGCTAAACACTCCGTGTAATATTACAGTCAAACATCATAATGCAAATGATGTCTTAACCCTTTTTCTTTTTTTTTTTTTGGCAATTGAGGCATATAAGTCGATGTAGCAAGATTCAGTTCTAAATTTTTGTAACAATTTTGAAAAATTTTACCGGTTAAGCTAATTAAAGCTATGAGTACATTAAATTTTAGGTTTAATTATCGTCCCCGATTGCATTATATTGTTTCATGTAAATTGTGTGGATTTTTAAAAAGGGGGGGGGGGGGTACGGACTAAACTCTCCTTATTATAAATTACGATGAAAATACAGAAGGATACACAGGAAAAAACATTCACAAGGAGAGAAAAAGAGATAAAATACAC
