# Supplementary material for: Enhancing cold tolerance through HubZIP6-mediated CBF activation and salicylic acid signaling in pitaya
Source: Mol Hortic. 2026 Sep 3;6:64. doi: 10.1186/s43897-026-00258-3 (PMC13540873; doi:10.1186/s43897-026-00258-3)
Supplement: Supplementary file 3 [file 43897_2026_258_MOESM3_ESM.docx]

**Supplementary Table 2-1** *Cis*-element analysis of the promoter of *HuCBF1*

| **Site names** | **Site** | **Motif sequences** | **The function of the motif** |
| --- | --- | --- | --- |
| MBS  Gap-box  TC-rich repeats  MYC  **G-box**  TGACG-motif  TCA-element  ARE  W box  TATC-box  MYB  ABRE  TC-rich repeats  CGTA/G-motif | 977(+), 1690(+)  1037(+)  1130(-)  27(+), 329(+),  349(-), 1395(-), 1883(+)  909(-), 1037(-)  1419(-)  1420(-), 1636(-), 1810(-)  1809(-), 1418(-)  926(-)  314(-), 1934(+)  1008(+), 1136(+), 1931(+)  326(-), 332(+), 798(-)  286(+)  74(+), 205(+), 798(-)  1420(-), 1636(-), 1810(-)  1421(+), 1637(+), 1811(+)  1634(+)  1130(-),  659(+), 1350(+), 651(-)  1160(+), 1424(+), 1640(+)  1790(+), 1813(+), 188(-)  361(-), 577(-) | CAACTG  CAAATGAA(A/G)A  GTTTTCTTAC  CATTTG, CAATTG  CATGTG  CATTTG  TAACACGTAG  CACGTG  ACACGTGT  TGACG  CCATCTTTTT  AAACCA  TTGACC  TATCCCA  CAACAG  CACGTG  ACGTG  GACACGTGGC  GTTTTCTTAC  CGTA  CGTG | MYB TF binding sites  part of a light responsive element  defense and stress responsiveness  MYC binding site  light responsiveness  MeJA-responsiveness  salicylic acid responsiveness  essential for the anaerobic induction  WRKY binding site  gibberellin-responsiveness  MYB binding site  abscisic acid responsiveness  defense and stress responsiveness  NAC binding site  NAC binding site |

**Supplementary Table 2-2** *Cis*-element analysis of the promoter of *HuCBF2*

| **Site** | **Motif sequences** | **The function of the motif** |
| --- | --- | --- |
| 630(+), 945(-),  1258(+), 1291(+)  1284(+)  1017(-), 1446(-)  1107(+), 1698(-), 1863(-)  1698(-), 1863(-)  1699(+), 1864(+)  500(-), 771(+)  995(+), 1222(+), 1266(+)  68(+)  285(+)  516(-), 646(-)  602(-), 721(+), 754(-), 1309(-)  917(-), 1822(-)  259(-), 292(+), 464(+), 1356(-)  643(-), 1086(-), 1442(+)  1457(-), 1683(-)  72(+), 398(+), 812(+), 881(+)  1341(+), 1745(+), 1793(+)  1983(+), 1928(-)  134(+), 377(+), 1699(+)  1840(+), 1864(+), 135(-)  456(-), 1182(-) | TAACTG  CAACTG  CAACCA  **CACGTG**  CACGTG  CAACTG  CACGTG  ACGTG  GGTTAA  TTGACC  AAACCA  GACACGTATG  CGTCA  TAATTACTC  CCGAAA  CATGTG CATTTG  CAATTG  CGTA  CGTG | Involved in drought-inducibility  antistress response element  Abscisic acid response element  light responsive element  light responsive element  part of a light responsive element  WRKY binding site  stress response element  light responsiveness  MeJA-responsiveness  wound-responsive element  ethylene-responsive element  **low-temperature responsiveness**  Anti-drought, anti-freeze, and anti-cold element  NAC binding site  NAC binding site |

**Supplementary Table 2-3** *Cis*-element analysis of the promoter of *HuCBF3*

| **Site names** | **Site** | **Motif sequences** | **The function of the motif** |
| --- | --- | --- | --- |
| **G-box**  TGA-element  **LTR**  TC-rich repeats  MYC  ABRE  CGTCA-motif  TGACG-motif  MYB  TATC-box  W box  CGTA/G-motif | 1427(+), 1428(-)  865(+)  200(+)  1137(-)  188(+), 269(+)  665(-), 646(+)  1428(-), 1428(+)  1734(+)  1734(-)  81(+)  530(+), 1862(+)  734(-)  1538(+)  191(+), 844(-)  365(+), 1430(+)  151(-), 486(-)  1026(+), 1767(+), 571(-) | **CCACGTAA**, TACGTG  AACGAC  CCGAAA  ATTCTCTAAC  CATTTG,  CATGTG, CAATTG  TACGTG, CACGTA  CGTCA  TGACG  TAACCA  CAACCA  TAACTG  TATCCCA  TTGACC  CGTA  CGTG | light responsiveness  auxin-responsive element  **low-temperature responsiveness**  defense and stress responsiveness  **Anti-drought, anti-freeze, and anti-cold element**  Abscisic acid response element  MeJA-responsiveness  Involved in drought-inducibility  gibberellin-responsiveness  WRKY binding site  NAC binding site  NAC binding site |
